# Prediction of Collision Cross-Section Values for Extractables and Leachables from Plastic Products

**DOI:** 10.1021/acs.est.2c02853

**Published:** 2022-06-22

**Authors:** Xue-Chao Song, Nicola Dreolin, Elena Canellas, Jeff Goshawk, Cristina Nerin

**Affiliations:** † Department of Analytical Chemistry, Aragon Institute of Engineering Research I3A, CPS-University of Zaragoza, Maria de Luna 3, 50018 Zaragoza, Spain; ‡ Waters Corporation, Altrincham Road, SK9 4AX Wilmslow, U.K.

**Keywords:** ion mobility, collision cross-section, plastic products, extractables, leachables, machine learning

## Abstract

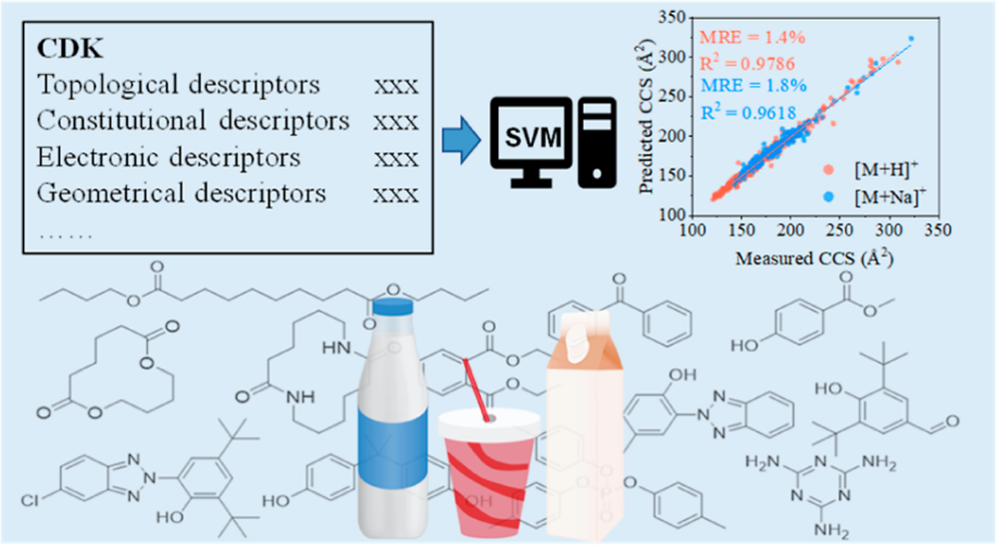

The use of ion mobility separation (IMS) in conjunction with high-resolution mass spectrometry has proved to be a reliable and useful technique for the characterization of small molecules from plastic products. Collision cross-section (CCS) values derived from IMS can be used as a structural descriptor to aid compound identification. One limitation of the application of IMS to the identification of chemicals from plastics is the lack of published empirical CCS values. As such, machine learning techniques can provide an alternative approach by generating predicted CCS values. Herein, experimental CCS values for over a thousand chemicals associated with plastics were collected from the literature and used to develop an accurate CCS prediction model for extractables and leachables from plastic products. The effect of different molecular descriptors and machine learning algorithms on the model performance were assessed. A support vector machine (SVM) model, based on Chemistry Development Kit (CDK) descriptors, provided the most accurate prediction with 93.3% of CCS values for [M + H]^+^ adducts and 95.0% of CCS values for [M + Na]^+^ adducts in testing sets predicted with <5% error. Median relative errors for the CCS values of the [M + H]^+^ and [M + Na]^+^ adducts were 1.42 and 1.76%, respectively. Subsequently, CCS values for the compounds in the Chemicals associated with Plastic Packaging Database and the Food Contact Chemicals Database were predicted using the SVM model developed herein. These values were integrated in our structural elucidation workflow and applied to the identification of plastic-related chemicals in river water. False positives were reduced, and the identification confidence level was improved by the incorporation of predicted CCS values in the suspect screening workflow.

## Abstract 2

Small molecules in plastics may migrate into the environment, leading to harmful impacts for the environment and human health. Collision cross-section (CCS) provided by ion mobility separation is helpful for the identification of small molecules. Here, a highly accurate CCS prediction model has been developed, and CCS values of thousands of chemicals associated with plastics have been predicted.

## 1 Introduction

Plastics play an important role in our daily life as they are used in a variety of materials, including packaging, building and construction materials, transportation, and electrical and electronic components.^1^ It has been reported that up to 2015, approximate 6300 million metric tons of plastic waste was generated of which only 9% was recycled. The remaining plastic waste was either incinerated, accumulated in landfills, or disposed of in natural environments.^2^ The impact of plastic waste on the environment and, subsequently, human health is of great concern due to the release of microplastics^3−5^ and low-molecular-weight (MW) chemicals.^6−9^ During the production of plastics, a variety of additives are incorporated into the polymeric formulations to enhance favorable characteristics and extend service life. Commonly used additives include plasticizers, flame retardants, lubricants, antioxidants, and UV stabilizers.^7^ Such additives have been detected in indoor dust,^10,11^ airborne particulate matters,^12−14^ waste water,^15^ soils,^16^ and rivers and oceans.^17−19^ Plastic products have become an important source of contaminants in aquatic and terrestrial environments.

In addition to the known substances included during the production of plastic materials, non-intentionally added substances (NIASs) can also occur. Typical NIAS include impurities, oligomers, and degradation products of material components,^20^ For example, organophosphate esters can result from the oxidation of organophosphite antioxidants in plastics and have been detected in indoor dust.^21,22^ If plastic products are made from recycled plastics, NIAS can also include contaminants resulting from the previous use of the material or from the recycling process itself.^23^ In recent years, the presence of perfluoroalkyl substances in plastic products has also attracted the attention of food safety and environmental authorities.^24−26^

The complete structural elucidation of extractables and leachables from plastics is a challenging process due to the complexity of the matrix. In recent years, ion mobility separation (IMS) coupled to high-resolution mass spectrometry (HRMS) has emerged as a promising tool for analyzing complex samples.^27−31^ IMS can separate molecules based on their shape, size, and charge.^32^ Collision cross-section (CCS), derived from IMS, is a physicochemical property of ions and is related to the chemical structure and three-dimensional conformation of the molecules.^32^ In addition, since CCS measurements are independent from chromatographic and mass spectrometric conditions, as well as the sample matrix,^33^ they can be used as an additional molecular identifier to increase the specificity and identification confidence. Celma et al.^34^ showed that CCS of imazalil was not affected by the sample matrix, whereas the retention time (RT) deviations ranged from 0.14 to 0.30 min; the consistent CCS values provided an extra point for unknown identification. In addition, incorporation the CCS values into the annotation process can help reduce false positive identifications^35^ and enable structural isomers to be separated and identified.^36,37^

Experimental CCS values of reference standards are often measured in order to confirm compound identification by comparing them to CCS values of candidate compounds in qualitative analyses. Although public CCS databases of pesticides,^38,39^ drugs,^40^ steroids,^41^ mycotoxins,^42^ and chemicals in plastic food packaging^43^ have been established, there remain many compounds that are not included in such libraries. As a matter of fact, many experimental CCS values of chemicals in plastics are not available due to the unavailability or high price of commercial standards. In this case, theoretical CCS values can be alternatives to be used for suspect and untargeted screening analysis. Several public CCS machine learning prediction tools have appeared in recent years, such as MetCCS,^44^ AllCCS,^35^ CCSondemand,^45^ CCSbase,^46^ and DeepCCS.^47^ Some laboratories have also developed their own CCS prediction tools for specific classes of compounds, such as pesticides,^38^ phenolics,^48^ and drugs.^49^ Many CCS values, belonging to different chemical classes, can provide a high structural diversity, and as such, the developed model can provide satisfactory prediction results for diverse chemical classes. At the time of writing, there are 3539, 7325, 7405, and 2439 CCS values in the data sets used by AllCCS, CCSondemand, CCSbase, and DeepCCS, respectively.

In a previous study,^43^ 635 CCS values derived from 488 standards associated with plastic packaging were used to develop a support vector machine (SVM) model to predict CCS values. The CCS values of 92.6% of protonated molecules were predicted with an error of less than 5%. The CCS values of some halogenated compounds were inaccurately predicted due to the lack of halogenated compounds in the training set. Consequently, in this study, additional experimental CCS values of molecules related to plastics have been collected from the literature, with the aim of achieving more accurate CCS prediction for chemicals found in plastics. The effect of different molecular descriptors (MDs) and algorithms on the accuracy of the CCS prediction were also explored. Following optimization and external validation, the model was used to predict CCS values of molecules in two plastic-related databases: the Chemicals associated with Plastic Packaging Database (CPPdb)^50^ and the Food Contact Chemicals Database (FCCdb).^51^ FCCdb also contains many plastic-related chemicals since approximately 37% of food contact materials (FCMs) are made from plastics.^52^ The two databases were subsequently converted into screening libraries, containing the predicted CCS values, and used for the suspect screening of plastic-related chemicals in river water.

## 2 Materials and Methods

### 2.1 CCS Data Collection and Processing

A total of 2145 experimental traveling wave CCS (^TW^CCS_N2_) and drift tube CCS (^DT^CCS_N2_) values were collected from seven recent publications,^27,29,38,39,43,53,54^ of which 1425 and 720 CCS values were for [M + H]^+^ and [M + Na]^+^ ions, respectively (Table S1). The CCS values in the publication of Song and co-workers^43^ were experimentally measured by injecting standards of chemicals associated with plastic food packaging. Four of the publications^29,38,39,53^ include CCS values mainly for pesticides and pharmaceuticals found in environmental studies. The CCS values in these four databases were used in this study because pesticides are an important type of NIAS in plastic materials, especially those made from the recycled plastics.^55^ Additionally, many pesticides contain halogens in their structure, as such, the predictions of CCS values for halogenated compounds will be more accurate by including the pesticides in the CCS data set. The last two publications^27,54^ mainly contain CCS values for organophosphorus flame retardants, compounds with a phosphate structure, which are common additives used in plastic materials. Since only three organophosphorus flame retardants were included in the previous self-built CCS database,^43^ the addition of the CCS values from these two publications significantly expanded the chemical diversity of the current study.

CCS values for some compounds appeared in more than one publication. In such cases, the CCS data were rationalized as follows:

(1) Chemical information retrieval: information including the compound identifier (CID), monoisotopic mass, molecular formula, canonical SMILES, and InChIKey of each CCS record was retrieved from PubChem using the R package *webchem.*(56)
(2) Calculation of median CCS values for duplicated records: in the cases where different names were used for the same compound in the different publications, the InChIKey was used as a unique identifier. The median and relative standard deviation (RSD) of multiple CCS values were calculated, and the median CCS values were used in the model.

A total of 1721 CCS values were retained after the consolidation of duplicate records, which included 1076 CCS values for [M + H]^+^ ions and 645 CCS values for [M + Na]^+^ ions. In consolidated data, the CCS values of 248 [M + H]^+^ ions (23.0%) and 72 [M + Na]^+^ ions (11.2%) were median values of multiple CCS records. The CCS data set rationalization was performed using the R package *tidyverse*,^57^ and the chemical class of each compound contributing to the model was obtained from ClassyFire.^58^

### 2.2 Calculation and Selection of Molecular Descriptors

MDs play a crucial role in the prediction of CCS values. In this work, three types of MDs were calculated using OCHEM^59^ and ChemDes.^60^ More information about MDs is shown in Supporting Information.

Descriptors that have a constant value or very few unique values relative to the number of samples have variance values equal or close to zero. Such descriptors contain little information and were considered less important for the model and excluded from the data set. Correlation coefficients (*r*) between individual MDs and CCS were subsequently calculated, and only the MDs for which *r* > 0.6 were retained. The remaining descriptors were auto-scaled to normalize the effect of the magnitude. The alvaDesc MDs were further rationalized by considering Extreme Gradient Boosting (XGBoost) importance. In XGBoost, the contribution of each variable to the model is calculated with respect to the number of times the variable is selected for splitting, weighted by the squared improvement to the model as a result of each split. The variable importance is then averaged across all the decision trees within the model.^61^ In this study, the alvaDesc MDs accounting for 99 and 95% of the total XGBoost importance were retained.

### 2.3 Development of the CCS Prediction Model

For both [M + H]^+^ and [M + Na]^+^ ions, the data were randomly divided into training and testing sets in the ratio of 7:3. The training set was used for the calibration and optimization of the model, and the testing set was used for the external validation. The comparison of CCS prediction accuracy between various models (models developed with different algorithms and MDs in this study as well as public CCS prediction tools) was based on the testing set data. The R code for model building was provided in GitHub (https://github.com/songxuechao/plasticCCS).

In addition to the CCS data and descriptors, the machine learning algorithm employed was another important factor, affecting the predictive performance of the model. In this study, two algorithms that are often used for CCS prediction were compared: XGBoost and SVM. XGBoost is an optimized distributed gradient boosting library designed to be highly efficient and flexible^62^ and was used to develop CCSondemand.^45^ The XGBoost model tuning consisted of 576 combinations of five important model parameters: eta (0.01, 0.05, 0.1, 0.3), max_depth (3, 5, 7), min_child_weight (1, 3, 5), subsample (0.6, 0.7, 0.8, 0.9), and colsample_bytree (0.6, 0.7, 0.8, 0.9). All combinations were evaluated using the training data set by a 10-fold cross validation. The optimal value of the *nrounds* parameter which controls the maximum number of iterations was returned using the minimized root-mean-square error of cross validation (RMSECV). Finally, the XGBoost model was built using the training data set with the optimized combination of parameters using the R package *xgboost*. The importance of MDs in the model was also calculated.

SVM is also a commonly used machine learning algorithm and has previously been used for the prediction of CCS values.^44,63^ In this study, SVM with the radial basis function kernel was used to build the model. Two important hyperparameters were optimized in order to get accurate predictions: cost of constraints violation (*C*) and gamma (γ). The *C* parameter trades off the predictive performance of the training set against the model’s margin, while the γ parameter defines how far the influence of a single training example reaches. Eight groups of *C* values (0.001, 0.005, 0.01, 0.025, 0.05, 0.1, 0.25, 0.5)/*N*_MD_ (i.e. number of MDs) and nine γ values (2^0^ to 2^8^) formed 72 parameter combinations, which were then evaluated using 10-fold cross validation on the training set. The parameter combination providing the minimum RMSECV was used in the SVM model using the R package *e1071*.

The performance of the models was assessed by comparing the following parameters: the coefficient of determination of the prediction (*R*_p_^2^), the root-mean-square error of the prediction (RMSEP), the median relative error (MRE), and the percentage of molecules with relative deviations from experimental CCS values of less than 2, 3, and 5%.

The prediction performance of our model was compared to three publicly available CCS prediction tools: CCSondemand (https://ccs.on-demand.waters.com) from Broeckling and co-workers,^45^ AllCCS (http://allccs.zhulab.cn) from Zhu lab,^35^ and CCSbase from Xu lab (http://ccsbase.net).^46^

### 2.4 Prediction of CCS Values for Compounds in CPPdb and FCCdb

The CPPdb consists of 4283 substances associated with plastic food packaging. The data set was rationalized by removing the metals and salts together with any substances with same InChIKey (replicates). Finally, only substances with a neutral mass between 50 and 1200 were retained. After following this procedure, 2883 substances from the CPPdb were retained. The FCCdb data set was also rationalized using the procedure described above, leading to 6508 substances retained in data set. The CCS values of the compounds retained from the databases were then predicted using the model that yielded the best performance in this study. Meanwhile, the chemical space covered by CPPdb, FCCdb, and our collected molecules was compared.

### 2.5 Application of Predicted CCS Values to the Analysis of Plastic-Related Chemicals in Ebro River Water

2L of surface water were sampled from the Ebro River near the urban areas of Zaragoza, Spain. The river water was stored in an amber glass bottle and treated on the day of collection, using the previously developed procedures.^30^ The final samples were analyzed using a Vion IMS-QTof mass spectrometer. The detailed procedures of sample treatment and operating conditions of the Vion are given in the Supporting Information. The features (*m*/*z*_RT_CCS pairs), obtained from Vion IMS-QTof, were then screened against two plastic-related databases, CPPdb (2883 compounds) and FCCdb (6508 compounds), containing *m*/*z* values, adducts, and predicted CCS values. The *m*/*z* deviations of the measured values were less than 5 ppm as for CCS deviation, the filter setting was based on its prediction accuracy.

## 3 Results

### 3.1 CCS Data Set

A total of 1076 and 645 CCS values were collated for [M + H]^+^ and [M + Na]^+^ adducts, respectively. CCS values ranged from 118.6 to 332.2 Å^2^ for the [M + H]^+^ data and from 134.7 to 321.9 Å^2^ for the [M + Na]^+^ data. Using ClassyFire,^58^ the compounds were categorized into 10 super classes for the [M + H]^+^ adduct and 11 super classes for the [M + Na]^+^ adduct. The principal super classes assigned were benzenoids, organoheterocyclic compounds, lipids and lipid-like molecules, and organic acids and derivatives (Figure S1). Benzenoids include compounds commonly detected in plastics such as phthalate-based plasticizers, antioxidants, bisphenols, primary aromatic amines, and pesticides.

248 and 72 duplicate CCS values were found for [M + H]^+^ and [M + Na]^+^ adducts, respectively, across the seven publications, and the RSDs of the measurements are shown in Figure S2. The RSD variation is less than 2% for 89.1% (221/248) of the [M + H]^+^ adducts of the molecules and 95.8% (69/72) of the [M + Na]^+^ adducts. Consequently, there are 27 and 3 CCS values with RSDs higher than 2% for the [M + H]^+^ and [M + Na]^+^ adducts, respectively, and the measurements contributing to these values are summarized in Tables S2 and S3. The majority of CCS values with RSDs greater than 2% were obtained from the publications of Bijlsma et al. (2017),^38^ Celma et al. (2020),^29^ and Regueiro et al. (2016).^39^ It appears that pesticide and drug-like compounds are more likely to produce a high variation of CCS values. Such compounds include picoxystrobin, acetopromazine, prochloraz, and oxadixyl, with the variation of the CCS measurements for the last two compounds being more than 20 Å^2^. The limit of CCS reproducibility, presence of protomers, and inconsistent CCS calibration across different instrument systems are three possible sources of deviations in CCS measurements. A more detailed explanation is given in the Supporting Information.

**Figure 1:**
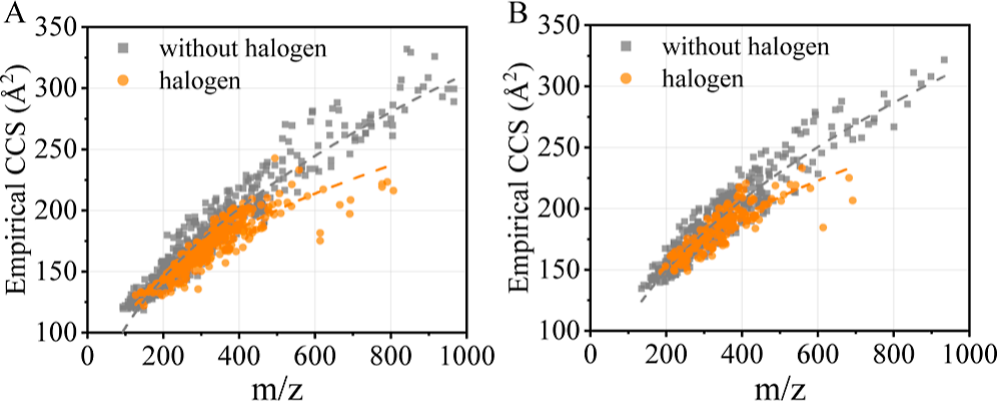
Empirical CCS vs *m*/*z* for (A) [M + H]^+^ and (B) [M + Na]^+^.

CCS is a value related to the size, shape, and charge of a molecule and understandably, CCS is also strongly correlated with the *m*/*z* value of a compound.^27,31,41,54^ The correlation between *m*/*z* and the CCS value of the compounds considered in this study is shown in Figure 1. In general, the relationship between *m*/*z* and CCS can be described by a power regression model. The inclusion of more halogenated compounds in this study (a total of 302 and 149 halogenated molecules were included for [M + H]^+^ and [M + Na]^+^ adducts, respectively), highlighted a distinct difference in their *m*/*z* and CCS relationship when compared to the relationship for non-halogenated compounds. The halogenated compounds tended to have smaller CCS values for a given *m*/*z*. It is believed that halogens have a lower atomic radius per atomic mass unit in comparison to other elements, such as C, H, O, and N. The partially orthogonal structural information provided by CCS is discussed in the Supporting Information.

Some CCS values collated in this study were measured using drift tube IMS (DTIMS),^27^ and deviations between ^TM^CCS_N2_ and ^DT^CCS_N2_ have previously been observed.^53^ Since accurate CCS values are fundamental to obtain a reliable CCS prediction model, the ^DT^CCS_N2_ values were compared to ^TM^CCS_N2_ values available in the literature (Tables S4 and S5). 16 ^TM^CCS_N2_ values were found in the literature that could be directly compared to ^DT^CCS_N2_ values, and most of these values were for compounds in the types of plasticizers and organophosphorus flame retardants. Table S4 shows that 81.3% of the values agree to within 2% and the deviations ranged from 0.11% (for atrazine) to 2.88% (for tri-*n*-butyl phosphate) with an average of 1.15%. In the case of the [M + Na]^+^ adduct, 75.0% of the values agree to within 2%, and the deviations ranged from 0.15% (for di-*n*-butyl phosphate) to 4.23% [for mono(2-ethylhexyl) adipate], with an average of 1.32%. The median of the ^TM^CCS_N2_ and ^DT^CCS_N2_ values was used when building the model to reduce any outlier measurements arising from the use of different IMS technologies.

### 3.2 Selection and Weighting of Molecular Descriptors

The selection of MDs can reduce training time, simplify the prediction model, and avoid overfitting; however, it is possible that meaningful information can also be lost, leading to a decrease in accuracy. For this reason, it is necessary to achieve a balance between the simplicity and accuracy of the model.

The numbers of MDs retained after each step of variable selection are shown in Figure S3, and the comparison of the model performance before and after variable selection is presented in Figure S4 and Tables S6–8. For alvaDesc MDs, the first 316 and 72 descriptors accounted for 99 and 95% of the total importance for [M + H]^+^ adducts. When the number of MDs was decreased from 1528 to 72, both the SVM and XGBoost models showed a slight decrease in the performance. The *R*_P_^2^ of the SVM model decreased from 0.9802 to 0.9737, RMSEP increased from 4.47 to 5.43, and MRE increased from 1.50 to 1.52%. Considering that the model was significantly simplified and the performance was still acceptable, the 72 most important alvaDesc MDs were selected for the [M + H]^+^ adduct data. In the case of the [M + Na]^+^ adduct CCS predictions, the models based on the first 193 MDs showed a comparable performance with the models built on 1361 MDs. Therefore, the 193 most significant MDs were selected for [M + Na]^+^ adduct data.

On determining the descriptors using CDK and RDKit, after the elimination of MDs that show low correlation with CCS (*r* < 0.6), 84 and 65 CDK descriptors and 33 and 27 RDKit descriptors were retained for [M + H]^+^ adducts and [M + Na]^+^ adducts, respectively; they were not filtered further. Table S7 shows that 84 CDK MDs can provide accurate prediction results for [M + H]^+^ adducts. A remarkable reduction in the performance of the model was observed for [M + Na]^+^ adducts, when the number of MDs was reduced from 207 to 65. Therefore, 84 and 207 CDK MDs were selected for the [M + H]^+^ and [M + Na]^+^ adducts, respectively. In the case of RDKit, 33 and 125 MDs were retained for [M + H]^+^ and [M + Na]^+^ adducts, respectively, based on the performance of the model (Table S8).

### 3.3 Model Performance

**Figure 2:**
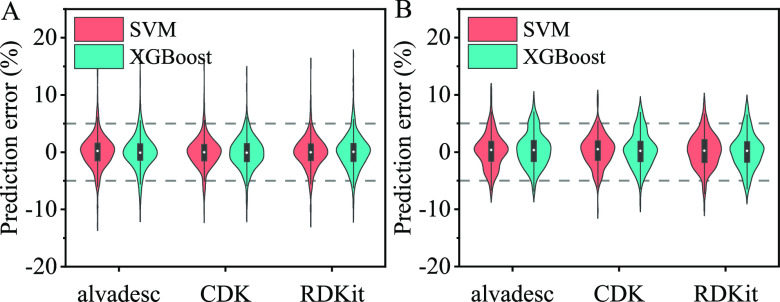
Violin-plot illustrating the prediction errors of the SVM and XGBoost models using different sets of descriptors: (A) [M + H]^+^ and (B) [M + Na]^+^.

After dividing the collated CCS values into a training data set and a testing data set, 329 and 181 CCS values were included in the testing set for [M + H]^+^ and [M + Na]^+^ adducts, respectively. For each adduct, six CCS prediction models were developed based on the combinations of two algorithms (XGBoost and SVM) and three types of MDs (alvaDesc, CDK, and RDKit). The distribution of prediction errors and model parameters for each model are shown in Figure 2 and Table 1, respectively. In the case of the [M + H]^+^ adducts, more than 90% of molecules showed prediction errors within 5% for all six models. The SVM-based model in conjunction with the CDK descriptors provided the best predictive performance. *R*_P_^2^ and MRE were 0.9786 and 1.42%, respectively, and more than 93 and 64% of molecules had prediction errors of less than 5 and 2%, respectively. This model also provided a better predictive performance for the [M + Na]^+^ adduct with more than 95 and 58% molecules having prediction errors of less than 5 and 2%, respectively. The results also show that the model for [M + H]^+^ adducts should use a different set of descriptors to those used for the model for [M + Na]^+^ adducts, implying that a unique CCS prediction model should be developed for each adduct. This is highlighted by the studies of Bijlsma et al. (2017),^38^ in which a single set of descriptors was used for the CCS prediction of all positive ions and demonstrated that the CCS values predicted for [M + Na]^+^ adducts were less accurate in general.

**Table 1:** Performance of the Models Developed Using Different Descriptors and Algorithms

adducts	descriptor	algorithm	*R*_p_^2^	RMSEP	<2%	<3%	<5%	MRE (%)
[M + H]^+^	alvaDesc	SVM	0.9737	5.43	61.7	79.0	91.8	1.52
		XGBoost	0.9727	5.53	61.7	75.7	90.6	1.44
	CDK	SVM	**0.9786**	**4.90**	**64.7**	**82.7**	**93.3**	**1.42**
		XGBoost	0.9765	5.14	59.6	78.7	94.2	1.61
	RDKit	SVM	0.9772	5.09	63.8	79.6	93.0	1.46
		XGBoost	0.9700	5.80	58.1	74.2	90.3	1.58
[M + Na]^+^	alvaDesc	SVM	0.9570	5.83	54.1	67.4	90.1	1.81
		XGBoost	0.9593	5.76	52.5	72.9	89.0	1.88
	CDK	SVM	**0.9618**	**5.53**	**58.0**	**74.6**	**95.0**	**1.76**
		XGBoost	0.9555	5.95	53.0	68.5	90.1	1.81
	RDKit	SVM	0.9511	6.18	49.2	72.9	90.1	2.01
		XGBoost	0.9577	5.82	53.6	69.1	87.8	1.81

In comparison to our previous study,^43^ a more accurate prediction of CCS values for [M + Na]^+^ adducts is achieved here. The value of RMSEP decreased from 8.2 to 5.5 Å^2^, the percentage of molecules with prediction errors less than 5% increased from 81.3 to 95.0% and those with prediction errors less than 2% increased from 54.7 to 58%. Even though there is a dramatic improvement, the prediction of CCS values for [M + Na]^+^ adducts was still less accurate than that for [M + H]^+^ adducts. It is believed that the main reason for this is that the MDs are calculated from neutral molecules, and a sodium adduct can lead to a more diverse range of molecular conformations in 3D space compared to protonation.^38,41,64^ One way to improve the accuracy of CCS predictions would be to determine the descriptors for ionized molecules, rather than neutral molecules. However, such an approach is more complicated and computationally expensive, in addition, conformational analysis is always required before the calculation of the descriptors.^65^

The CCS prediction for halogenated molecules was also more accurate using the current SVM model compared to our previous study.^43^ 95.3% (81 out of 85 molecules) and 65.9% (56 out of 85 molecules) of protonated halogenated molecules had prediction errors of less than 5 and 2%, respectively. This compares to our previous study^43^ for which the percentages were only 86.7 and 40%, respectively. This significant improvement could be due to the additional halogenated molecules in the training set, which supports previous observations that structure similarity between predictions and the training set significantly affect the accuracy of CCS predictions.^35^ To further validate this conjecture, we excluded the 217 halogenated molecules from the training set for [M + H]^+^ adducts, leaving 530 non-halogenated molecules to rebuild the SVM model for the prediction of CCS values for molecules in the testing set. A comparison of CCS prediction results, with and without halogenated compounds in the training set, is shown in Figure S5. It is evident that upon excluding halogenated compounds from the training set, the prediction errors for the 244 non-halogenated compounds in the test data are similar to those generated when the halogens were included in the training data. However, the predicted CCS values of 85 halogenated compounds in the test data has significantly larger errors when the halogens were excluded from the training data: MRE increased from 1.46 to 1.87%, and the proportion of halogenated compounds with prediction errors <2% decreased from 65.9 to 54.1%. This confirms that the chemical diversity of training set is an important factor, which affects the prediction accuracy for the test data.

The protonated molecules for which the prediction error in the CCS value was greater than 5% were further investigated. The presence of protomers can lead to high CCS prediction errors. For example, two different CCS values (160.5 and 176.2 Å^2^) have been reported for acetopromazine in previous studies,^29,38^ the predicted CCS value of 179.8 Å^2^ matched well with the CCS value of the more extended protomer. Similar behavior was also observed in the work of Zhou et al.^44^ More discussions are given in the Supporting Information.

Through the comparison of the six models and the comparison with our previous study,^43^ the SVM model based on CDK MDs provided the most accurate predictions. The chemical diversity of the training set seems to be a more crucial factor for CCS prediction than descriptors and algorithms. The possibility of multiple protomers is another important factor, affecting the accuracy since only one predicted CCS value can currently be determined for a given adduct by machine learning models. Besides, we opted to use SVM due to its easy configuration with few hyperparameters, as well as its ability to provide reproducible prediction results.

### 3.4 Comparison between the SVM Model and Public CCS Prediction Tools

The outcomes from the SVM model based on CDK MDs were compared to those from three publicly available CCS prediction tools: CCSondemand, AllCCS, and CCSbase. The distributions of the prediction errors for all models are illustrated in Figure S6, and the corresponding MRE for each chemical class is shown in Figure S7.

The CCS values of 65 and 74% of protonated molecules were predicted with an error of less than 2% by SVM and CCSondemand, respectively. More than 93% of protonated molecules has prediction errors less than 5% for both models. CCSondemand was trained by approximately 7325 experimental ^TW^CCS_N2_ values obtained from 3775 compounds.^45^ The training data set contains CCS values of chemicals found in plastic food packaging and pesticides, so when the CCS values of such molecules are predicted by CCSondemand, one would expect smaller prediction errors. The predictive capabilities of AllCCS and CCSbase were not as good as those for SVM and CCSondemand for the compounds considered in this study. This is possibly due to the dissimilarity of the structures of chemicals in plastics and the molecules used in the training sets of AllCCS and CCSbase.

The results for [M + Na]^+^ adducts showed that the SVM model gave more accurate predictions than the other tools. The enhanced performance of SVM is possibly due to the higher number of MDs used in this model (*n* = 207) as only 15 MDs were used in AllCCS.^35^ More detailed comparison is shown in the Supporting Information.

The AllCCS tool is also based on the SVM algorithm and CDK MDs; however, there are two main differences between AllCCS and our model: the training data and the number of MDs. In order to investigate which factor leads to the significantly different prediction results between AllCCS and our model, we built a SVM model based on our CCS training data and the 15 MDs used for AllCCS and compared their prediction results for the testing set to those obtained from our original model and AllCCS (Table S9). For both [M + H]^+^ and [M + Na]^+^ adducts, less accurate prediction results were obtained from SVM models based on 15 MDs than with our original SVM model. MRE values increased from 1.4 to 1.6%, for [M + H]^+^ adducts and 1.8 to 2.1% for [M + Na]^+^ adducts. The results from the SVM model based on 15 MDs and the AllCCS tool using the same MDs but different training data can be seen in Table S9. AllCCS shows significantly larger prediction errors, with MRE values of 2.2% for [M + H]^+^ adducts and 3.3% for [M + Na]^+^ adducts. These results show that the data used to train the model have a greater effect on the prediction accuracy of the model than the MDs.

These results show that, when compared to other available prediction tools, the SVM model based on the CDK MDs can improve the prediction of CCS values, especially for sodiated molecules. The CCS values for the [M + H]^+^ and [M + Na]^+^ adducts of the molecules in CPPdb and FCCdb were subsequently predicted by the SVM model developed here. The two databases were then transformed into screening libraries, which were used for the suspect screening of plastic-related chemicals in Ebro River water.

### 3.5 Plastic-Related Chemicals Tentatively Identified in Ebro River Water

Approximately 95% of predicted CCS values (93.3% for [M + H]^+^ adducts and 95.0% for [M + Na]^+^ adducts) are within 5% deviation with respect to experimental values. Thus, the tolerance for CCS deviations was set as 5% in the suspect screening of plastic-related chemicals in Ebro River water. Two main aspects of using predicted CCS values in the identification of unknowns were investigated: reducing the number of false positives and increasing the confidence level of identified compounds. The river water samples were screened against 9391 compounds in CPPdb and FCCdb to search for plastic-related chemicals. The number of candidates with and without the confirmation of CCS values was compared. The addition of the CCS filter decreased the number of candidate compounds from 376 to 204 (45.7%).

A total of 98 plastic-related chemicals were tentatively identified in the Ebro River surface water samples from the CPPdb and FCCdb databases, of which 26 compounds were confirmed using reference standards. The tentatively identified compounds consisted of 12 plasticizers, 10 flame retardants, 6 antioxidants, 9 slip agents, 10 dyes, and 26 surfactants (including glycol and glycerol derivatives). NIAS were also detected in Ebro River water, including the ethylene terephthalate cyclic trimer, a common oligomer of polyethylene terephthalate,^66^ and bisphenol A bis(2,3-dihydroxypropyl) ether, a hydrolysis product of bisphenol A diglycidyl ether.^67^ Detailed information about the identified compounds is available in the Supporting Information.

**Figure 3:**
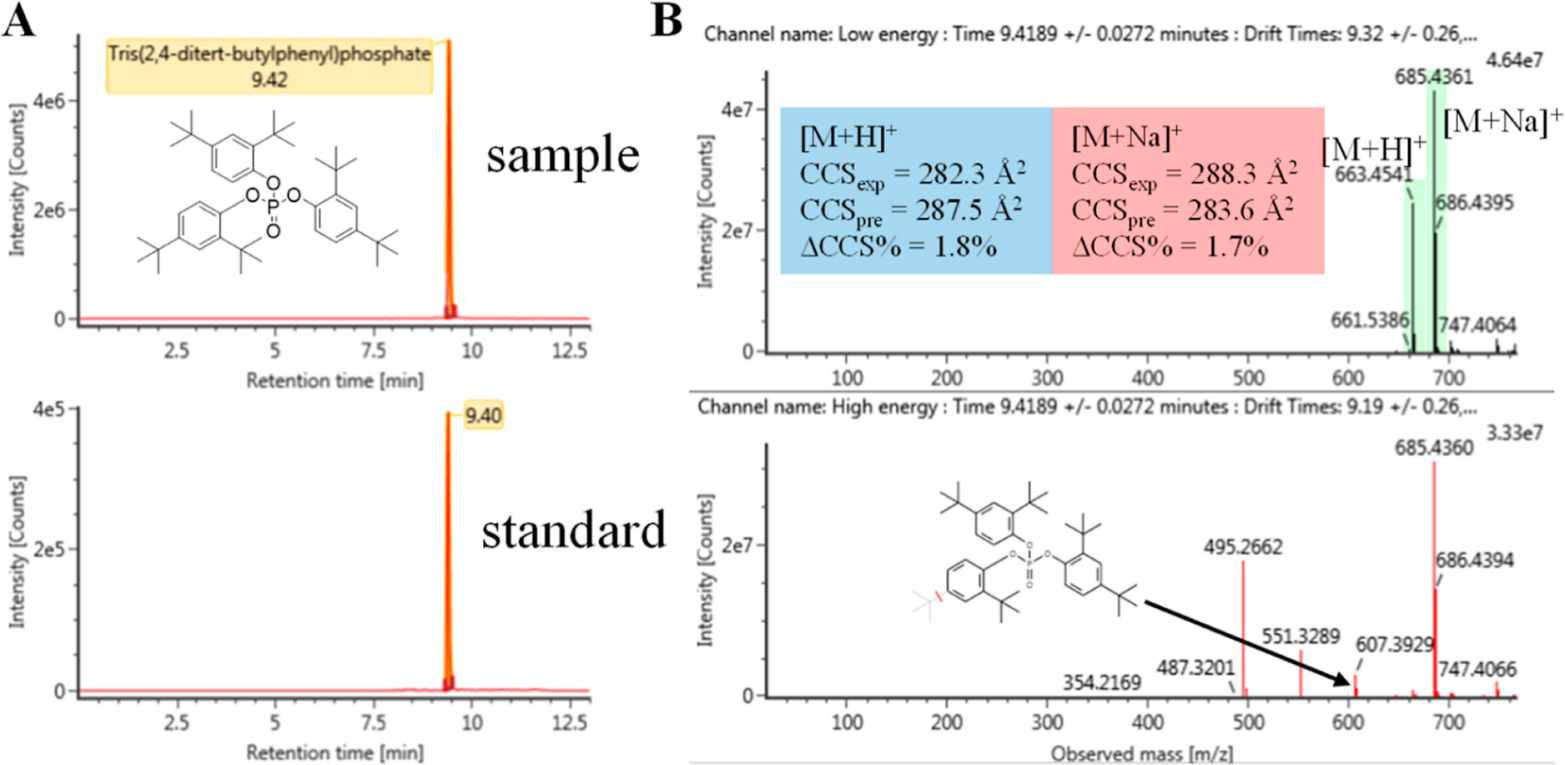
Identification of tris(2,4-ditert-butylphenyl)phosphate. (A) Extracted ion chromatograms from the sample and standard, (B) low- and high-energy spectra, fragment assignment, comparison between experimental and predicted CCS values.

The most abundant compound detected in the Ebro River water samples was tris(2,4-ditert-butylphenyl)phosphate. This is a degradation product of Irgafos 168 (a commonly used phosphite antioxidant in plastics).^68^ Previous studies have shown that tris(2,4-ditert-butylphenyl)phosphate was an abundant contaminant in indoor dust^21^ and fine particulate matter.^14^ The predicted CCS values for tris(2,4-ditert-butylphenyl)phosphate had deviations less than 2% versus the experimental values (Figure 3).

The benefit of predicted CCS values in identification of unknowns is more relevant either when the analyte is at low concentration levels or the reference standard is not available. 1,4,7-Trioxacyclotridecane-8,13-dione is a reaction product from adipate plasticizer/adipate acid and ethylene glycol, and its molecular structure and mass spectra are shown in Figure S8. The figure shows the isotopic pattern for the [M + H]^+^ adduct was indistinct, and no fragment ions were observed in the high-energy spectrum for the compound, possibly due to the low concentration and ineffectual assignment of fragment ions to the respective precursor ions. A fragment ion at *m*/*z* value 155.0699 was observed in low-energy spectrum, which corresponds to the loss of the ethylene glycol unit. CCS deviation for the [M + H]^+^ and [M + Na]^+^ adducts of 1,4,7-trioxacyclotridecane-8,13-dione was 0.4 and −2.1%, respectively. In this case, even though no abundant fragmentation information was obtained for 1,4,7-trioxacyclotridecane-8,13-dione, the combination of RT, *m*/*z* and CCS contribute to a reliable identification.

In some cases, even when the analyte is at a high concentration in the sample, fragments ions may still not be assigned in the high-energy mass spectrum, as a result of rigid structures of less labile molecules. An example of this is given in Figure S9, in which the mass spectra of Antiblaze V6, a flame retardant in plastics, are shown. The fragment ions observed in the high-energy spectrum are in low abundance, and substructures of the parent molecule could not be assigned. The predicted CCS value (207.9 Å^2^) had a 3.5% deviation when compared to the experimental value (215.2 Å^2^). Additionally, an experimental CCS value (211.4 Å^2^) for Antiblaze V6 was found in the literature^27^ and has a deviation of −1.8% from our experimental value. It is not possible to confirm the identification of this compound due to the lack of a reference standard; however, the comparison between predicted and experimental CCS values, the *m*/*z* values, and the characteristic chlorine isotopic pattern provides high confidence for the assignment.

False positive assignments have been observed for which the tolerances of *m*/*z* error <5 ppm and CCS deviation <5% are satisfied. For example, the ion with *m*/*z* 327.0785 and CCS 169.9 Å^2^ at a RT of 9.42 min is a good match for triphenyl phosphate. However, the reference standard was detected with a RT of 6.50 min, thereby showing this assignment to be a false positive. The addition of RT predictions may be able to eliminate this kind of false positives, as shown by previous studies.^67,68^

## 4 Discussion

### 4.1 Suitability of Combining Both ^DT^CCS_N2_ and ^TW^CCS_N2_ Values in the Model

CCS values measured using DTIMS can differ from those measured using traveling wave IMS (TWIMS) platforms.^53^ Therefore, the suitability of combining both ^DT^CCS_N2_ and ^TW^CCS_N2_ values in the CCS prediction model was investigated. 16 compounds have both ^DT^CCS_N2_ and ^TW^CCS_N2_ values for both [M + H]^+^ and [M + Na]^+^ adducts (Tables S4 and S5). Measurements of ^DT^CCS_N2_ alone are present for 39 [M + H]^+^ adducts and 65 [M + Na]^+^ adducts. Of these, 27 ^DT^CCS_N2_ values for [M + H]^+^ adducts and 51 ^DT^CCS_N2_ values for [M + Na]^+^ adducts are present in the training data. The ^DT^CCS_N2_ values were removed from the training data, the SVM models were rebuilt, and their performance was compared to the original SVM models (Table S10). The prediction accuracy of the CCS values for the [M + H]^+^ adducts remained similar to the original results; however, the predicted CCS values for the [M + Na]^+^ adducts were less accurate. RMSEP increased from 5.5 to 5.8 Å^2^, and the proportion of compounds with prediction errors was <2% decreased from 58.0 to 55.8%. The reduction in the prediction accuracy upon removing the ^DT^CCS_N2_ values from the training data is probably due to the reduction in the diversity of chemical structures. The ^DT^CCS_N2_ values were mainly for organophosphate flame retardants and phthalate monoesters, both of which are additives commonly used in plastics.^11,13^

It should be noted that in this study, the differences between ^DT^CCS_N2_ and ^TW^CCS_N2_ values are relatively small. Higher CCS deviations were observed between ^TW^CCS_N2_ values from different laboratories than between ^DT^CCS_N2_ and ^TW^CCS_N2_ values (Tables S2–S5). Based on these observations, we decided to use both ^DT^CCS_N2_ and ^TW^CCS_N2_ values in the training data of the model.

### 4.2 Weighting and Collinearity of CDK MDs

The important CDK MDs for the prediction of the CCS values are shown in Figures S10 and S11, and a brief description of these important MDs is also given in Table S11 and Supplemental Results and Discussion. The effect of collinearity between CDK MDs was investigated by building models that omitted highly correlated MDs. The variance inflation factor (VIF) is a measure of the correlation between MDs with higher values indicating greater correlation. Two models were built to study collinearity of MDs, one for which MDs with a VIF value greater than 50 were excluded and one for which MDs with a VIF value greater than 20 were excluded. A comparison of the predictive performance between the two new models and the original model is shown in Figure S12. The figure shows that for [M + H]^+^ adducts, 33 MDs with a VIF value below 50 were retained and 24 MDs with VIF value below 20 were retained. The reduction in the number of the MDs slightly decreased the prediction accuracy of both the SVM and XGBoost models. Similar behavior was observed when the same procedure was applied to the models for the [M + Na]^+^ adduct. Since the models built with 84 and 207 CDK MDs for the [M + H]^+^ and [M + Na]^+^ adducts, respectively, provide more accurate predictions, and the complexity of the model was still deemed to be acceptable, the number of MDs was not reduced in the final models.

### 4.3 Approaches to Improve the Prediction Accuracy

There are several ways in which the accuracy of CCS predictions could potentially be improved. First, more experimental CCS values can be collected for the training set to increase the chemical diversity and universality of the model. A total of 17 and 15 chemical super classes are considered in CCSondemand and AllCCS respectively, while in this study, only 10 and 11 super classes are covered by 1076 and 645 CCS values for [M + H]^+^ and [M + Na]^+^ adducts, respectively. Table S12 presents the 50 compounds in CPPdb and FCCdb that were not covered by the chemical space of our collected CCS records. Generally, these compounds have high molecular mass and contain long linear-chain structures.

Second, MDs based on ionized molecules could improve predictions, especially for [M + Na]^+^ adducts. There is a much bigger difference between the structural conformation of sodiated and neutral molecules than there is between protonated and neutral molecules.^64^ This makes it difficult to obtain accurate predicted CCS values for sodiated molecules when the descriptors are derived from neutral molecules. Taking into account that deriving MDs from ionized molecules is time-consuming and complex, and as such, MDs derived from neutral molecules are probably sufficiently accurate for [M + H]^+^ adducts. In the study by Gonzales et al. (2016),^48^ MDs of deprotonated phenolics were determined for a CCS prediction model, and 92.8% (52/56) of molecules was predicted within 5% of their measured values. In the present study, a similar proportion (93.3%) of protonated molecules was predicted with an error less than 5%, highlighting that MDs determined from neutral molecules are sufficient for the accurate prediction of CCS values of protonated molecules.

Third, improving the reproducibility of commercially available IMS devices such as TWIMS and DTIMS will lead to more precise and accurate CCS measurements, which, when used as inputs to prediction models, will improve the performance of the models. At the time of writing, commercial IMS devices have relatively low reproducibility, which makes it impractical to adopt an accuracy threshold lower a than 2% when matching measured CCS values to library values within a suspect screening workflow.^41,42,69^

### 4.4 Current Limitations and Future Prospects

In this study, the CCS prediction models were only built for positive ions. This is understandable to some extent as most additives in plastic products, such as plasticizers, antioxidants, flame retardants, photoinitiators, and slip agents, are detected in the positive ion mode.^20^ In some cases, compounds can be only detected, or show a higher response, in the negative ion mode. Such compounds include lubricants (lauric acid and oleic acid) and surfactants (perfluorooctanesulfonic acid and perfluorobutanesulfonic acid), which were detected in the Ebro River water samples using our in-house plastic additives library (see the Supporting Information). Therefore, a CCS prediction model for negative ions needs to be developed in the future. Additionally, the CCS prediction models developed herein are only available to a small set of privileged users, and work needs to be undertaken to develop them into an open-access tool.

Many emerging contaminants associated with plastics, such as tricaprin, polyethylene glycol, and polypropylene glycol oligomers, do not exist in the CPPdb and FCCdb databases. These compounds were detected at high abundance in the Ebro River water samples using our in-house plastic additives library. With the rapid growth of newly reported plastic-related chemicals, the CPPdb and FCCdb databases need to be continuously expanded and updated. The construction of an integrated plastic-related database containing name, adducts, *m*/*z* values, predicted CCS values, and predicted RTs will facilitate the identification of extractables and leachables from plastics in HRMS-based screening strategies.

In summary, the SVM model, based on CDK descriptors presented here, provided more accurate CCS predictions than the XGBoost algorithm and other descriptors. The CCS values of 93.3% [M + H]^+^ adducts and 95.0% [M + Na]^+^ adducts were predicted within 5% of their measured values. It has been shown that the chemical diversity of the training set appears to have more influence on the predictive performance than alternative algorithms and MDs investigated here. Indeed, CCS predictions for halogenated compounds were more accurate following the incorporation of more CCS records of halogenated compounds into the training set. Increasing the number of experimental CCS values and improving the reproducibility of CCS measurements seem to be two feasible ways to further increase the performance of prediction models. In future work, a CCS prediction model for negative ions will be developed, and work toward making all models open-access will be undertaken.

## Supporting Information Available

Chemical classes of collected molecules; RSDs of CCS values; variable selection processes; optimization of MDs; effect of halogenated compounds; comparison between SVM models to other public tools; identification of 1,4,7-trioxacyclotridecane-8,13-dione; mass spectra of Antiblaze V6; influential CDK descriptors; correlation between CCS and Atomic and Bond Contributions of van der Waals volume (VABC); effect of collinearity; collected experimental CCS values; CCS records with RSD higher than 2%; comparison between ^DT^CCS_N2_ and ^TW^CCS_N2_; optimization of alvaDesc, CDK, and RDKit descriptors; comparison with AllCCS; comparison of SVM models before and after excluding ^DT^CCS_N2_ values; calculation of MDs; river water treatment; conditions of Vion IMS-QTOF; sources of CCS deviations; reasons leading to high prediction errors; comparison between SVM to public tools; and important CDK descriptors (PDF)
Collected empirical CCS values (XLSX)
Plastic-related chemicals tentatively identified in Ebro River water (XLSX)

## Abbreviations

IMS: ion mobility separation
HRMS: high-resolution mass spectrometry
CCS: collision cross-section
SVM: support vector machine
MRE: median relative errors
CPPdb: Chemicals associated with Plastic Packaging Database
FCCdb: Food Contact Chemicals Database
MW: molecular weight
NIAS: non-intentionally added substances
PFAS: perfluoroalkyl substances
FCMs: food contact materials
^TW^CCS_N2_: traveling wave CCS
^DT^CCS_N2_: drift tube CCS
CID: compound identifier
RSDs: relative standard deviations
MD: molecular descriptor
*r*: correlation coefficients
XGBoost: extreme gradient boosting
RMSECV: root-mean-square error of cross validation
RBF: radial basis function
*C*: cost of constraint violation
γ: gamma
*R*_p_^2^: determination coefficient of prediction
RMSEP: root-mean-square error of prediction
RT: retention time
DTIMS: drift tube ion mobility separation
PET: polyethylene terephthalate
TWIMS: traveling wave ion mobility separation
VIF: variance inflation factor
PEG: polyethylene glycol
PPG: polypropylene glycol
