# Supplementary material for: Prediction of Collision Cross-Section Values for Extractables and Leachables from Plastic Products
Source: Environ Sci Technol. 2022 Jun 22;56(13):9463–73. doi: 10.1021/acs.est.2c02853 (PMC9261268; doi:10.1021/acs.est.2c02853)
Supplement: Supplementary file 1 — es2c02853_si_001.pdf [file es2c02853_si_001.pdf]

Supporting Information

Prediction of Collision Cross Section Values for Extractables and Leachables from Plastic Products

Xue-Chao Song,[†] Nicola Dreolin,[‡] Elena Canellas,[†] Jeff Goshawk,[‡] Cristina Nerin*,[†]

[†]Department of Analytical Chemistry, Aragon Institute of Engineering Research I3A, CPS-University of Zaragoza, Torres Quevedo Building, María de Luna 3, 50018 Zaragoza, Spain.

[‡]Waters Corporation, Altrincham Road, SK9 4AX Wilmslow, United Kingdom.

*Corresponding author: Cristina Nerin, Tel: +34 976761873; Email: cnerin@unizar.es.

Table of Contents:

Figure S1. Chemical classes of compounds for (A) $[M+H]^+$ and (B) $[M+Na]^+$.

Figure S2. Relative standard deviation (RSD) of collision cross section (CCS) values of identical molecules obtained from different instrument platforms and different laboratories, (A) $[M+H]^+$ and (B) $[M+Na]^+$.

Figure S3. The number of molecular descriptors retained after each step of variable selection.

Figure S4. Optimization of descriptors from alvaDesc (A), CDK (B) and RDKit (C).

Figure S5. Comparison of the CCS prediction accuracy including and excluding halogenated compounds from the training set for $[M+H]^+$ adducts. "Normal" represents the SVM model built with all 747 compounds in the training set; "Simulation" represents the SVM model built with only the 530 non-halogenated compounds in the training set; "non-halogen" and "halogen" represent 244 non-halogenated compounds and 85 halogenated compounds in testing set, respectively.

Figure S6. Violin-plot and bar-plot showing the comparison of the CCS predictions of the SVM model to other CCS prediction tools: (A) $[M+H]^+$; (B) $[M+Na]^+$.

Figure S7. Heat-map displaying median relative errors (MRE) of different chemical super classes obtained from the model presented here and other CCS prediction tools.

Figure S8. Identification of 1,4,7-trioxacyclotridecane-8,13-dione. (A) molecular structure and predicted CCS values; (B) extracted ion chromatogram from sample; (C) low and high energy spectra, fragment assignment.

Figure S9. Mass spectra and CCS values of Antiblaze V6.

Figure S10. Relative importance of the 20 most influential CDK descriptors for the prediction of CCS vlaues of $[M+H]^+$ adducts in XGBoost model.

Figure S11. Correlation between Atomic and Bond Contributions of van der Waals volume (VABC) and CCS values of $[M+H]^+$.

Figure S12. Comparison of CCS prediction accuracy between before and after excluding highly correlated descriptors, (A) $[M+H]^+$, CDK_84, CDK_33 and CDK_24 represent models

based on 84, 33 (VIF<50) and 24 (VIF<20) CDK descriptors; (B) $[M+Na]^+$, CDK_207 and CDK_101 represent models based on 207 and 101 (VIF<50) CDK descriptors.

Figure S13. Comparison of the chemical space of FCCdb, CPPdb and our collected CCS records.

Table S1. Experimental CCS values retrieved from scientific literatures.

Table S2. Compounds for which the relative standard deviation (RSD) of the CCS values of the $[M+H]^+$ adduct is higher than 2%.

Table S3. Compounds for which the relative standard deviation (RSD) of the CCS values of the $[M+Na]^+$ adduct is higher than 2%.

Table S4. Comparison between $^{DT}CCS_{N2}$ and $^{TW}CCS_{N2}$ for $[M+H]^+$.

Table S5. Comparison between $^{DT}CCS_{N2}$ and $^{TW}CCS_{N2}$ for $[M+Na]^+$.

Table S6. Optimization of alvaDesc descriptors.

Table S7. Optimization of CDK descriptors.

Table S8. Optimization of RDKit descriptors.

Table S9. Comparison of the prediction results obtained from SVM models using 84 and 207 descriptors in our study, SVM models using 15 selected descriptors in AllCCS, and AllCCS.

Table S10. Comparison of SVM models before and after excluding 27 and 51 $^{DT}CCS_{N2}$ values from the training set of $[M+H]^+$ and $[M+Na]^+$.

Table S11. Definition of 20 important CDK molecular descriptors.

Table S12. The 50 molecules in CPPdb and FCCdb that are not covered by the chemical space of the collected CCS records.

Supplemental Materials and Methods: Calculation of Molecular Descriptors**;** River Water Treatment; Conditions of Vion IMS-QTOF.

Supplemental Results and Discussion: Three possible sources of CCS deviations; Partially orthogonal molecular information provided by CCS; Reasons leading to high prediction errors; Comparison Between the SVM Model and Public CCS Prediction Tools; Weighting of CDK descriptors; Applicability of Our CCS Prediction Model.

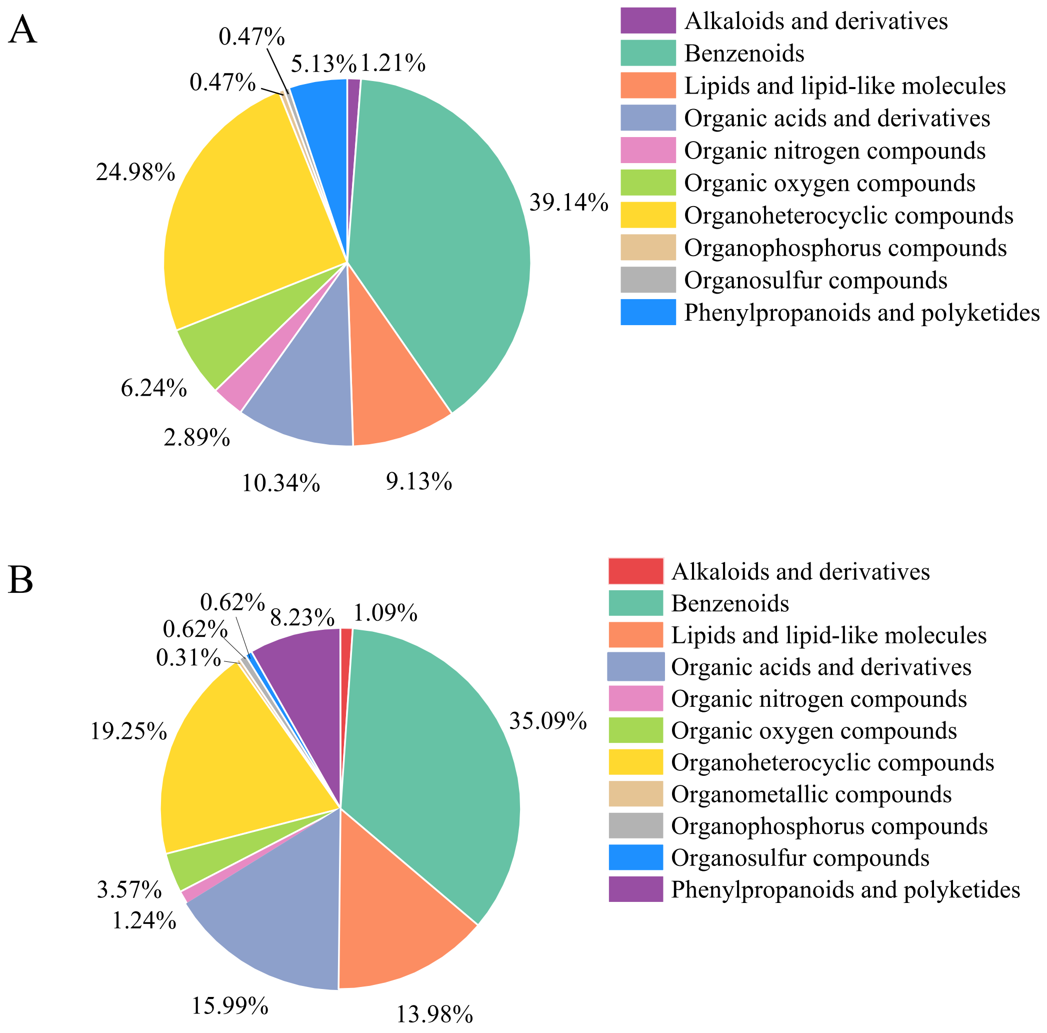

Figure S1. Chemical classes of 1076 compounds in $[M+H]^+$ (A) and 645 compounds in $[M+Na]^+$ (B).

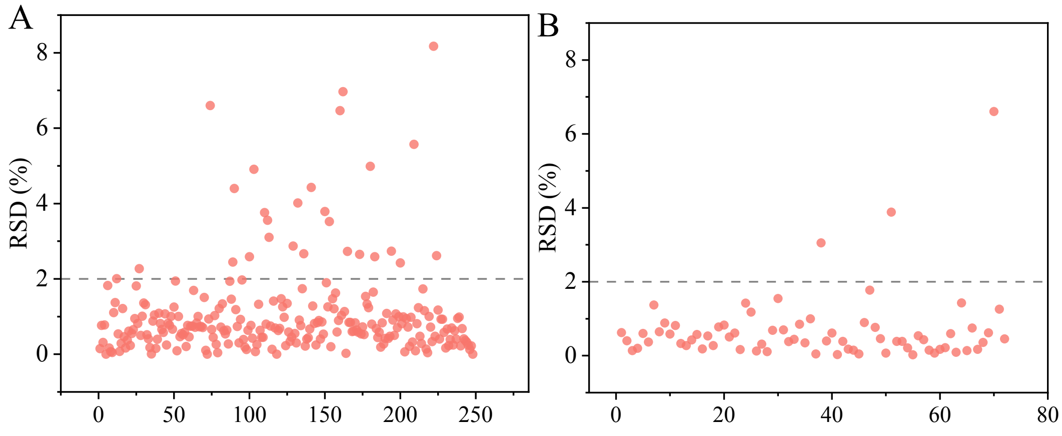

Figure S2. Relative standard deviation (RSD) of collision cross section (CCS) values of identical molecules obtained from different instrument platforms and different

laboratories, (A) $[M+H]^+$ and (B) $[M+Na]^+$.

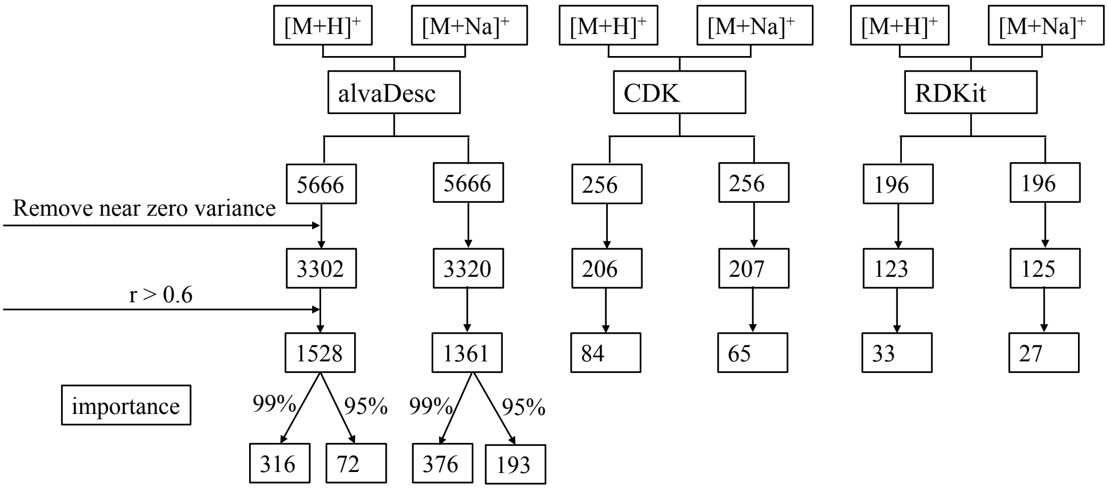

Figure S3. The number of molecular descriptors retained after each step of variable selection.

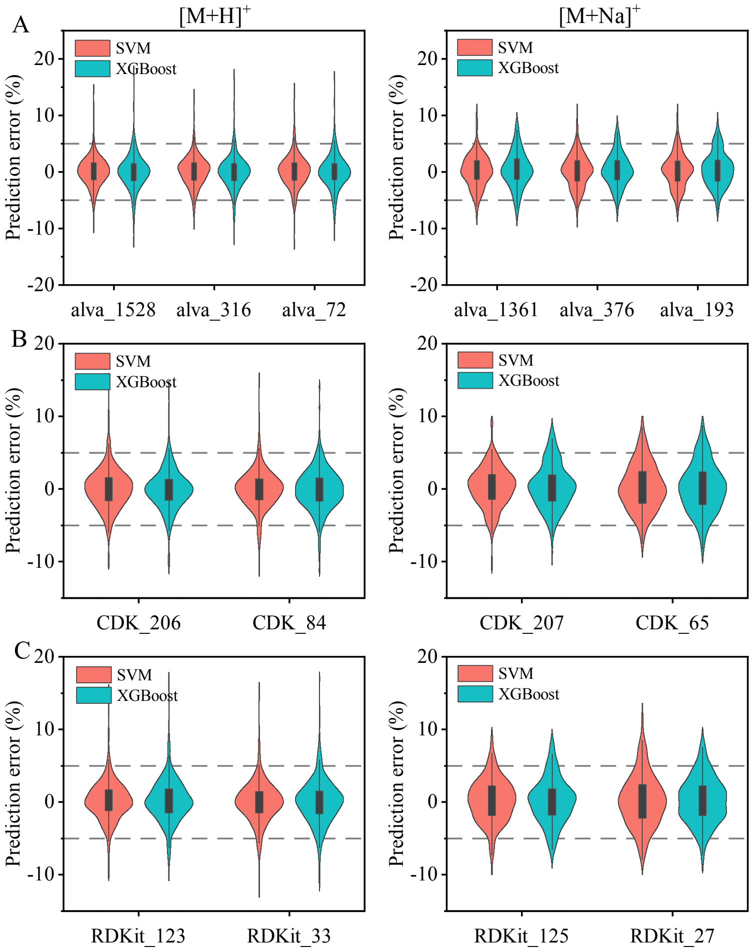

Figure S4. Optimization of descriptors from alvaDesc (A), CDK (B) and RDKit (C).

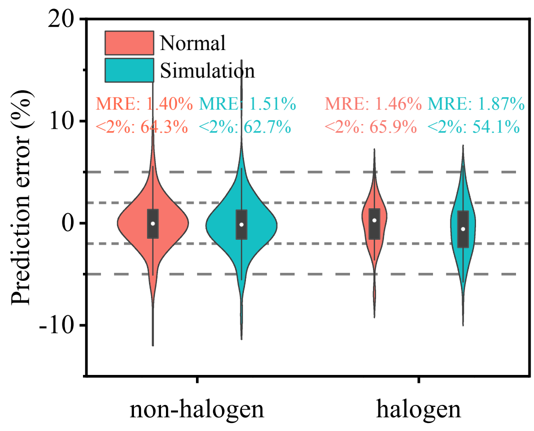

Figure S5. Comparison of the CCS prediction accuracy including and excluding halogenated compounds from the training set for $[M+H]^+$ adducts. “Normal” represents the SVM model built with all 747 compounds in the training set; “Simulation” represents the SVM model built with only the 530 non-halogenated compounds in the training set; “non-halogen” and “halogen” represent 244 non-halogenated compounds and 85 halogenated compounds in testing set, respectively.

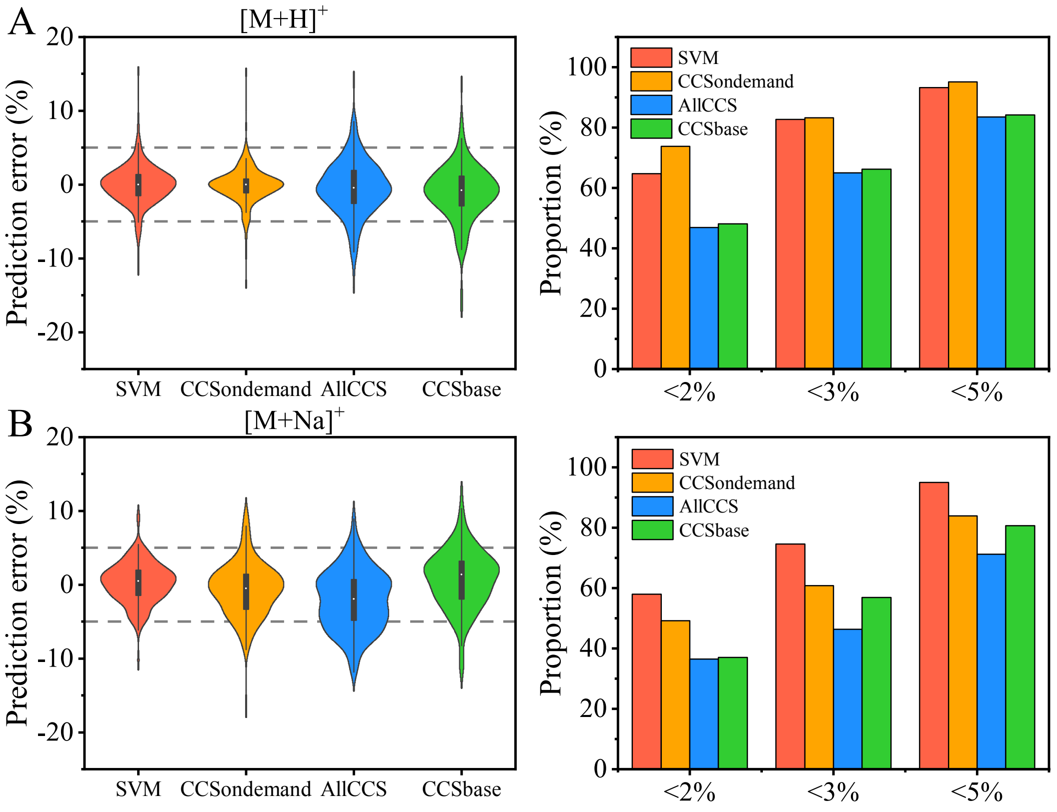

Figure S6. Violin-plot and bar-plot showing the comparison of the CCS predictions of the SVM model to other CCS prediction tools: (A) $[M+H]^+$; (B) $[M+Na]^+$.

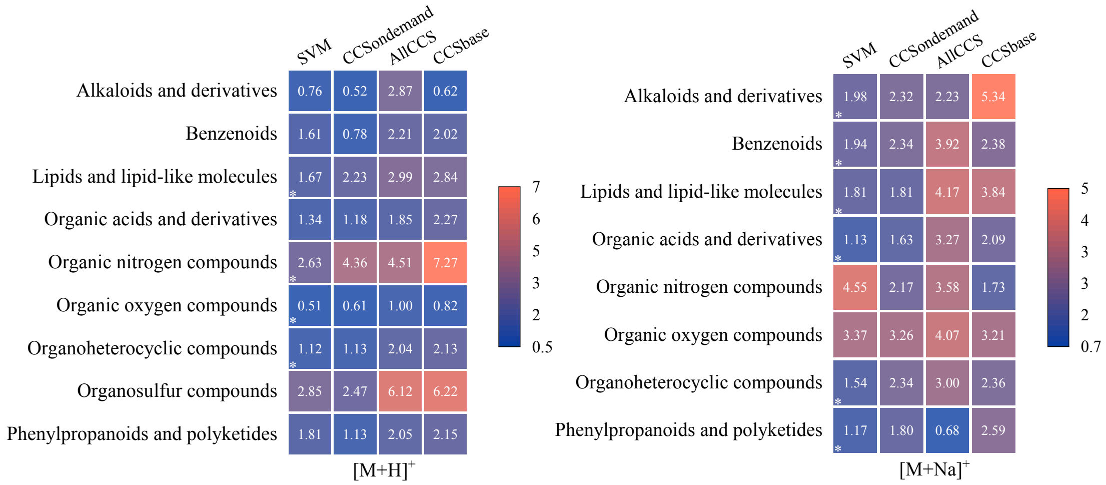

Figure S7. Heat-map displaying median relative errors (MRE) of different chemical super classes obtained from the model presented here and other CCS prediction tools.

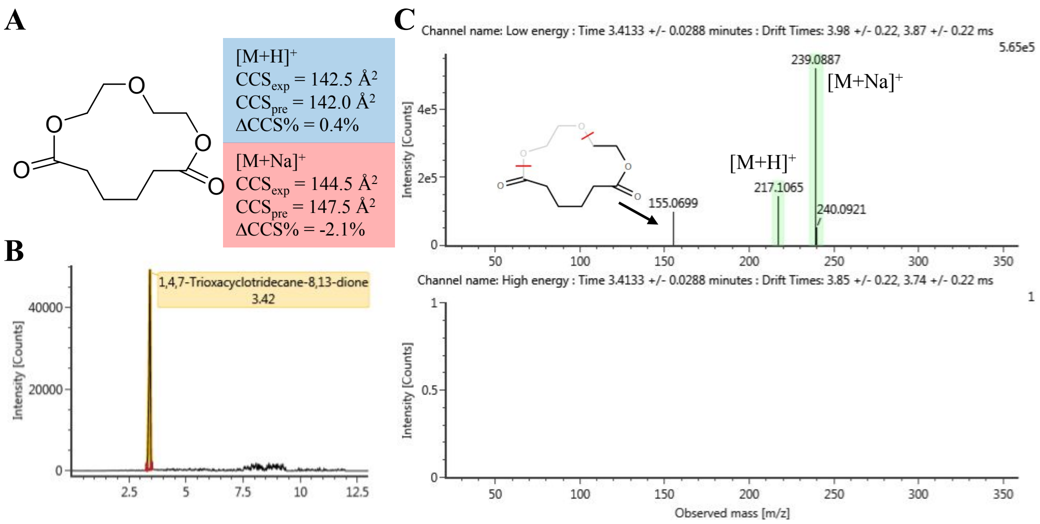

Figure S8. Identification of 1,4,7-trioxacyclotridecane-8,13-dione. (A) molecular structure and predicted CCS values; (B) extracted ion chromatogram from sample; (C) low and high energy spectra, fragment assignment.

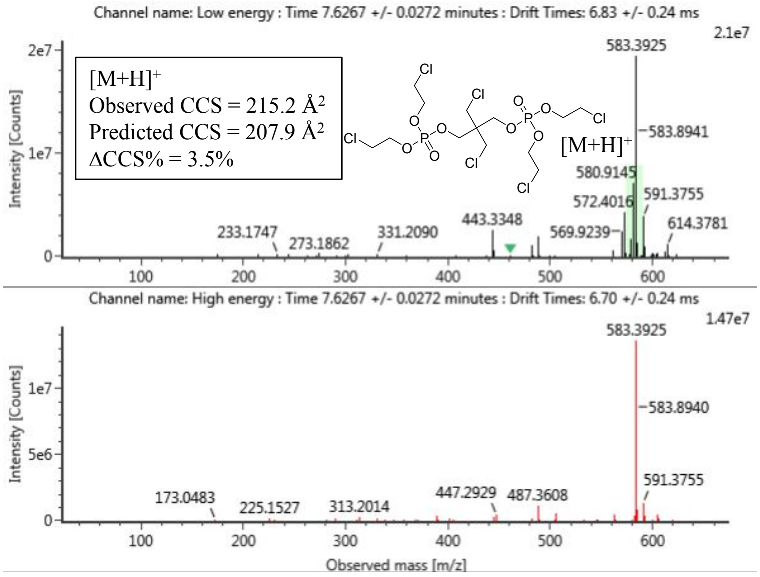

Figure S9. Mass spectra and CCS values of Antiblaze V6.

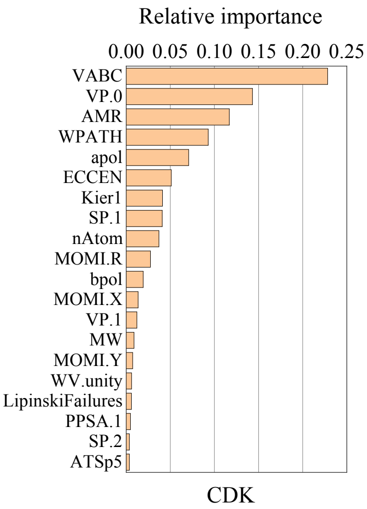

Figure S10. Relative importance of the 20 most influential CDK descriptors for the prediction of CCS vlaues of $[M+H]^+$ adducts in XGBoost model.

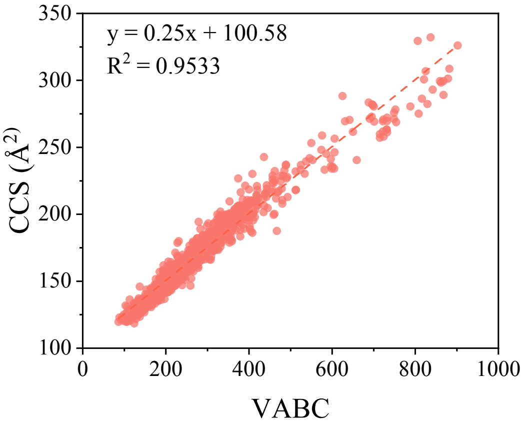

Figure S11. Correlation between Atomic and Bond Contributions of van der Waals volume (VABC) and CCS values of $[M+H]^+$.

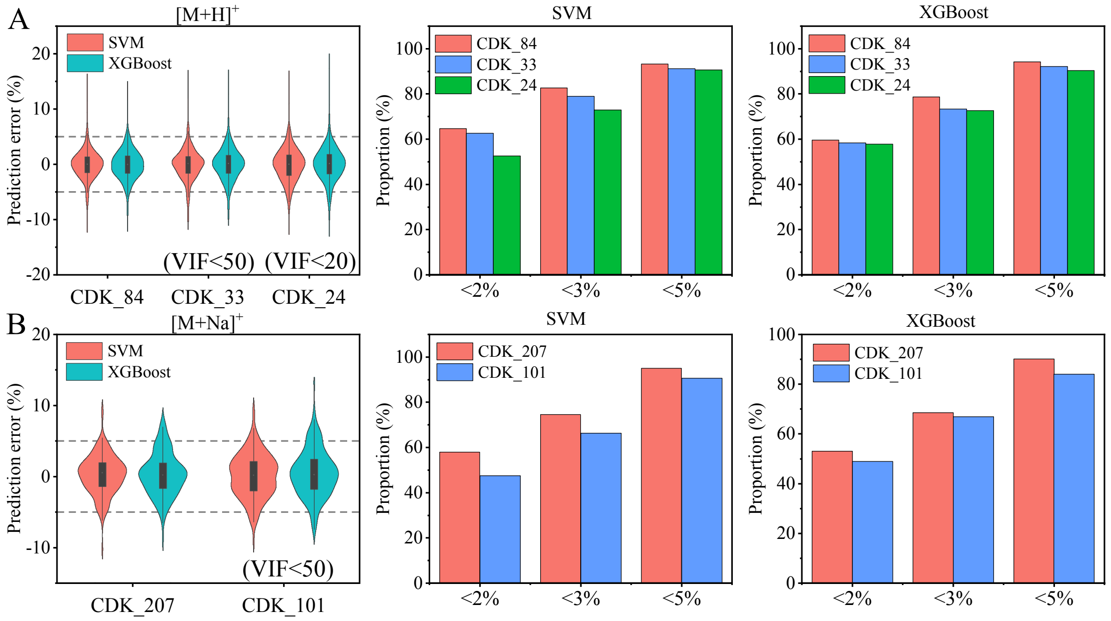

Figure S12. Comparison of CCS prediction accuracy between before and after excluding highly correlated descriptors, (A) $[M+H]^+$, CDK_84, CDK_33 and CDK_24 represent models based on 84, 33 (VIF<50) and 24 (VIF<20) CDK descriptors; (B) $[M+Na]^+$, CDK_207 and CDK_101 represent models based on 207 and 101 (VIF<50) CDK descriptors.

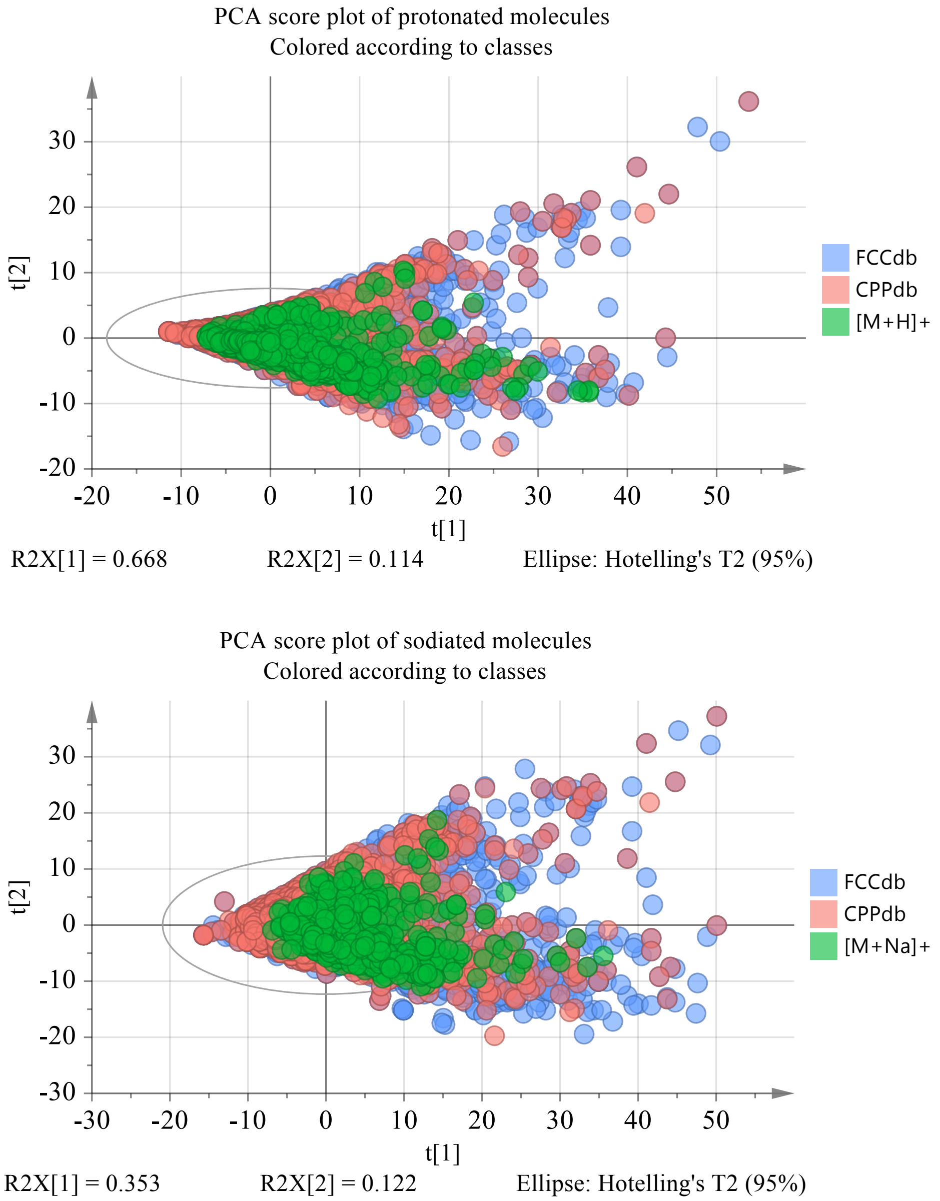

Figure S13. Comparison of the chemical space of FCCdb, CPPdb and our collected CCS records.

Table S1. Experimental CCS values retrieved from scientific literatures.

Compound type	Technology	$[M+H]^+$	$[M+Na]^+$	Reference
Chemicals in food plastic packaging, including antioxidant, plasticizers, UV absorbers, lubricants, and NIAS	$^{TW}CCS_{N2}$	401	272	[1]
Pesticides	$^{TW}CCS_{N2}$	205	0	[2]
Organic environmental pollutants, including illicit drugs, hormones, mycotoxins, new psychoactive substances, pesticides, and pharmaceuticals	$^{TW}CCS_{N2}$	460	243	[3]
Pesticides	$^{TW}CCS_{N2}$	177	34	[4]
Pesticides and pharmaceuticals	$^{TW}CCS_{N2}$	91	88	[5]
Contaminants of emerging concern in human matrix, including bisphenols, plasticizers, organophosphate flame retardants and triazoles	$^{DT}CCS_{N2}$	55	81	[6]
Pollutants in indoor dust: flame retardants, pesticides	$^{TW}CCS_{N2}$	36	2	[7]

Table S2. Compounds for which the relative standard deviation (RSD) of the CCS values of the $[M+H]^+$ adduct is higher than 2%.

CID	Name	CCS_1	CCS_2	CCS_3	Mean	RSD (%)
31357	Tributyl phosphate	171.53[1]	166.73[6]	-	169.13	2.01
5329	Sulfamethoxazole	151.7[1]	146.3[2]	152.61[3]	150.2	2.27
86222	Carfentrazone-Ethyl	179.54[3]	185.8[4]	-	182.67	2.42
2783	Clenbuterol	159.20[2]	164.81[3]	-	162.01	2.45
86369	Sulfentrazone	173.63[2]	180.1[4]	-	176.87	2.59
56208	Sarafloxacin	192.3[2]	202.0[2]	194.5[3]	196.28	2.59
2206	Phenazone	135.59[3]	140.7[5]	-	138.15	2.62
5094	Ronidazole	131.6[2]	136.63[3]	-	134.12	2.65
26879	Levamisole	138.2[2]	143.51[3]	-	140.86	2.67
3778	Propyphenazone	150.0[2]	155.9[5]	-	152.95	2.73
5280440	Tylosine	332.4[2]	319.79[3]	-	326.10	2.73
121858	Hydroxymetronidazole	128.2[2]	133.51[3]	-	130.86	2.87
3352	Fipronil	180.77[2]	181.45[3]	191[4]	184.41	3.10
4539	Norfloxacine	171[2]	179.74[3]	-	175.37	3.52
9576412	Fenpyroximate	215.89[2]	205.3[4]	-	210.60	3.56
3343	Fenoterol	180.4[2]	171.06[3]	-	175.73	3.76
4173	Metronidazole	124.2[2]	131.04[3]	-	127.62	3.79
26951	Ipronidazole	126.1[2]	133.47[3]	-	129.79	4.02
46783606	Clencyclohexerol	164.7[2]	175.28[3]	-	169.99	4.40
60651	Marbofloxacin	179.6[2]	191.21[3]	-	185.41	4.43
3090	Dimetridazole	116.4[2]	124.77[3]	-	120.59	4.91
5336	Sulfapyridine	142.3[2]	152.7[3]	-	147.5	4.99
108192	Epitestosterone glucuronide	204.7[3]	221.5[3]		213.09	5.57
11285653	Picoxystrobin	177.5[2]	194.5[4]	-	186.00	6.46
6077	Acetopromazine	160.5[2]	176.22[3]	-	168.36	6.60
73665	Prochloraz	161.68[2]	182.48[3]	183.3[4]	175.82	6.97
53735	Oxadixyl	158.81[3]	178.3[4]	-	168.56	8.18

Table S3. Compounds for which the relative standard deviation (RSD) of the CCS values of the $[M+Na]^+$ adduct is higher than 2%.

CID	Name	CCS_1	CCS_2	Mean	RSD (%)
20342	2-Ethylhexyl adipate	169.83[1]	177.32[6]	173.58	3.05
39042	Bezafibrate	198.51[3]	187.90[5]	193.21	3.88
45358380	lambda-Cyhalothrin	208.66[3]	190.04[3]	199.35	6.60

Table S4. Comparison between $^{DT}CCS_{N2}$ and $^{TW}CCS_{N2}$ for $[M+H]^+$ adducts.

No.	Name	CID	$^{DT}CCS_{N2}$[6]	$^{TW}CCS_{N2}$[1]	Differences (%)	Remarks
1	3,5-ditert-butyl-4-hydroxybenzaldehyde	73219	165.21	164.86	0.21	Degradation product of BHT
2	Atrazine	2256	149.53	149.70	0.11	Herbicide
3	Diazinon	3017	173.15	173.38	0.13	Insecticide
4	Benzotriazole	7220	122.42	123.49	0.87	UV absorbent
5	Di(2-ethylhexyl) phthalate	8343	211.00	213.33	1.10	Plasticizer
6	Diisononyl phthalate	590836	220.60	226.37	2.62	Plasticizer
7	Isodecyl diphenyl phosphate	34697	200.20	202.40	1.10	Plasticizer
8	Tributyl acetyl citrate	6505	199.82	195.99	1.92	Plasticizer
9	Antiblaze V6	92310	211.37	212.10	0.35	Flame retardant
10	Tri-n-butyl phosphate	31357	166.73	171.53	2.88	Flame retardant
11	Triphenyl phosphate	8289	174.74	170.00	2.71	Flame retardant
12	Tri-p-tolyl phosphate	6529	190.02	187.00	1.59	Flame retardant
13	Tris(1,3-dichloro-2-propyl) phosphate	26177	178.56	176.50	1.15	Flame retardant
14	Tris(2-butoxyethyl) phosphate	6540	196.44	198.30	0.95	Flame retardant
15	Tris(2-chloroethyl) phosphate	8295	151.31	150.40	0.60	Flame retardant
16	Tris(2-chloroisopropyl) phosphate	26176	161.66	161.87	0.13	Flame retardant

Table S5. Comparison between $^{DT}CCS_{N2}$ and $^{TW}CCS_{N2}$ for $[M+Na]^+$ adducts.

No.	Name	CID	$^{DT}CCS_{N2}$[6]	$^{TW}CCS_{N2}$[1]	Differences (%)	Remarks
1	Di(2-ethylhexyl) adipate	7641	218.46	218.84	0.18	Plasticizer
2	Di(2-ethylhexyl) phthalate	8343	215.33	217.47	0.99	Plasticizer
3	Di(2-ethylhexyl) terephthalate	22932	215.81	217.69	0.87	Plasticizer
4	Dibutyl sebacate	7986	193.48	191.61	0.97	Plasticizer
5	Diisodecyl phthalate	33599	226.42	232.18	2.54	Plasticizer
6	Diisononyl phthalate	590836	220.94	225.45	2.04	Plasticizer
7	Dimethyl sebacate	7829	159.71	160.41	0.44	Plasticizer
8	Mono(2-ethylhexyl) adipate	20342	177.32	169.83	4.23	Plasticizer
9	Mono(2-ethylhexyl) phthalate	20393	182.27	181.25	0.56	Plasticizer
10	Tributyl acetyl citrate	6505	205.77	205.28	0.24	Plasticizer
11	Diphenyl phthalate	6778	181.27	178.28	1.65	Plasticizer
12	2-Ethylhexyl diphenyl phosphate	14716	202.70	198.80	1.92	Flame retardant
13	Di-n-butyl phosphate	7881	167.54	167.79	0.15	Flame retardant
14	Tri-n-butyl phosphate	31357	184.54	184.16	0.21	Flame retardant
15	Tris(2-chloroethyl) phosphate	8295	161.39	157.89	2.17	Flame retardant
16	Tri-p-tolyl phosphate	6529	200.02	196.04	1.99	Flame retardant

Table S6. Optimization of alvaDesc descriptors.

Adducts	Descriptor	Algorithm	R^2_P	RMSEP	<2%	<3%	<5%	MRE
$[M+H]^+$	alva_1528	SVM	0.9802	4.71	65.3	81.2	94.8	1.50
		XGBoost	0.9724	5.56	60.5	76.3	90.6	1.48
	alva_316	SVM	0.9779	5.00	62.3	79.3	93.6	1.54
		XGBoost	0.9734	5.46	63.2	79.6	92.4	1.46
	**alva_72**	SVM	0.9737	5.43	61.7	79.0	91.8	1.52
		XGBoost	0.9727	5.53	61.4	75.7	90.6	1.44
$[M+Na]^+$	alva_1361	SVM	0.9629	5.44	53.0	72.9	92.8	1.86
		XGBoost	0.9511	6.20	51.4	67.4	86.2	1.95
	alva_376	SVM	0.9589	5.70	50.3	70.2	91.7	1.94
		XGBoost	0.9611	5.57	54.1	71.8	90.1	1.75
	**alva_193**	SVM	0.9570	5.73	54.1	67.4	90.1	1.81
		XGBoost	0.9593	5.76	52.5	72.9	89.0	1.88

Table S7. Optimization of CDK descriptors.

Adducts	Descriptor	Algorithm	R^2_P	RMSEP	<2%	<3%	<5%	MRE
$[M+H]^+$	CDK_206	SVM	0.9778	4.99	60.5	78.1	92.7	1.56
		XGBoost	0.9775	5.02	64.4	77.2	93.6	1.46
	**CDK_84**	SVM	0.9786	4.90	64.7	82.7	93.3	1.42
		XGBoost	0.9765	5.14	59.6	78.7	94.2	1.61
$[M+Na]^+$	**CDK_207**	SVM	0.9618	5.53	58.0	74.6	95.0	1.76
		XGBoost	0.9555	5.95	53.0	68.5	90.1	1.81
	CDK_65	SVM	0.9484	6.35	47.5	63.0	88.4	2.21
		XGBoost	0.9481	6.45	46.4	63.0	85.6	2.22

Table S8. Optimization of RDKit descriptors.

Adducts	Descriptor	Algorithm	R^2_P	RMSEP	<2%	<3%	<5%	MRE
$[M+H]^+$	RDKit_123	SVM	0.9787	4.90	62.0	78.7	94.5	1.44
		XGBoost	0.9744	5.36	59.9	74.5	92.4	1.61
	**RDKit_33**	SVM	0.9772	5.09	63.8	79.6	93.0	1.46
		XGBoost	0.9700	5.80	58.1	74.2	90.3	1.58
$[M+Na]^+$	**RDKit_125**	SVM	0.9511	6.18	49.2	72.9	90.1	2.01
		XGBoost	0.9577	5.82	53.6	69.1	87.8	1.81
	RDKit_27	SVM	0.9389	6.96	43.1	61.9	85.1	2.35

	XGBoost	0.9564	5.89	51.4	66.3	89.0	1.97

Table S9. Comparison of the prediction results obtained from SVM models using 84 and 207 descriptors in our study, SVM models using 15 selected descriptors in AllCCS, and AllCCS.

Adducts	Models	R^2_P	RMSEP	<2%	<3%	<5%	MRE (%)
$[M+H]^+$	SVM_84	**0.9786**	**4.90**	**64.7**	**82.7**	**93.3**	**1.42**
	SVM_15	0.9763	5.17	61.4	76.3	92.7	1.57
	AllCCS	0.9609	6.92	47.0	64.9	83.5	2.16
$[M+Na]^+$	SVM_207	**0.9618**	**5.53**	**58.0**	**74.6**	**95.0**	**1.76**
	SVM_15	0.9430	6.68	49.2	64.6	82.9	2.10
	AllCCS	0.9185	9.21	36.5	46.4	71.3	3.29

Table S10. Comparison of SVM models before and after excluding 27 and 51 $^{DT}CCS_{N2}$ values from the training set of $[M+H]^+$ and $[M+Na]^+$.

Adducts	Training set	R^2_P	RMSEP	<2%	<3%	<5%	MRE (%)
$[M+H]^+$	747	**0.9786**	**4.90**	**64.7**	**82.7**	**93.3**	**1.42**
	720	0.9785	4.91	64.1	82.1	93.3	1.40
$[M+Na]^+$	464	**0.9617**	**5.53**	**58.0**	**74.6**	**95.0**	**1.77**
	413	0.9572	5.80	55.8	72.9	91.7	1.78

Table S11. Definition of 20 important CDK molecular descriptors.

Descriptor name	Descriptor class	Definition
VABC	Geometrical Descriptor	Calculates van der Waals volume of molecules
VP.0, VP.1, SP.1, SP.2	Topological Descriptor	Evaluates the Kier & Hall Chi path indices of orders 0,1,2
AMR	Constitutional Descriptor	Calculates atom additive molar refractivity values as described by Ghose and Crippen
WPATH	Topological Descriptor	Calculates Wiener path number
apol	Electronic Descriptor	Calculates the sum of the atomic polarizabilities (including implicit hydrogens)
bpol	Electronic Descriptor	Descriptor that calculates the sum of the absolute value of the difference between

		atomic polarizabilities of all bonded atoms in the molecule (including implicit hydrogens)
ECCEN	Topological Descriptor	A topological descriptor combining distance and adjacency information
Kier1	Topological Descriptor	Calculates Kier and Hall kappa molecular shape indices
nAtom	Constitutional Descriptor	Descriptor based on the number of atoms of a certain element type
MOMI.R, MOMI.X MOMI.Y	Geometrical Descriptor	Descriptor that calculates the principal moments of inertia and ratios of the principal moments. Als calculates the radius of gyration
MW	Constitutional Descriptor	Descriptor based on the weight of atoms of a certain element type
WV.unity	Hybrid Descriptor	Holistic descriptors described by Todeschini et al.
LipinskiFailures	Constitutional Descriptor	Contains a method that returns the number failures of the Lipinski's Rule Of Five.
PPSA.1	Electronic Descriptor Geometrical Descriptor	A descriptor combining surface area and partial charge information
ATSp5	Topological Descriptor	The Moreau-Broto autocorrelation descriptors using polarizability

Note: information is from OCHEM (http://forum.ochem.eu/x/GgJr.html)

Table S12. The fifty molecules in CPPdb and FCCdb that are not covered by the chemical space of the collected CCS records.

Name	PubChem CID	Monoisotopic Mass	InChIKey
Polyoxyethylene (23) lauryl ether	2724258	1198.8013	IEQAICDLOKRSRL-UHFFFAOYSA-N
Pentaerythritol tetraoleate	6436503	1193.0548	QTIMEBJTEBWHOB-PMDAXIHYSA-N
Cetyl poly(oxyethylene) ether	2724259	1122.7853	NLMKTBGFQGKQEV-UHFFFAOYSA-N
2,2-bis[[3-(dodecylthio)-1-oxopropoxy]methyl]propane-1,3-diyl bis[3-(dodecylthio)propionate]	122423	1160.8179	VSVVZZQIUJXYQA-UHFFFAOYSA-N
Glyceryl tribehenate	62726	1059.0180	DMBUODUULYCPAK-UHFFFAOYSA-N
Homopolymer of glyceryl triester with 12-glycidyl-9-octadecenoic acid	58604493	1100.8467	ZFJYZDDXGKWNCH-UHFFFAOYSA-N
Glyceryl tri(12-acetoxystearate)	6451270	1064.8467	FNOXLRARSOMOQK-UHFFFAOYSA-N
Tris(dinonylphenyl) phosphite	74003	1066.9210	WRSPWQHUHVRNFV-UHFFFAOYSA-N

Pentaerythritol tetrakis(3-(3,5-di-tert-butyl-4-hydroxyphenyl)propionate)	64819	1176.7841	BGYHLZZASRKEJE-UHFFFAOYSA-N
Starch, hydrogen phosphate, 2-hydroxypropyl ether	24847848	1198.4140	DVROLKBAWTYHHD-UHFFFAOYSA-N
Octadecanoic acid, 12-hydroxy-, polymer with alpha-hydro-omega-hydroxypoly(oxy-1,2-ethanediyl)	121596032	948.6233	GJHBWXDMLVECGP-UHFFFAOYSA-N
Sorbitol trioleate	129772621	1022.7997	IEFFZOKIZPKMOG-RJUOWQTLSA-N
2-Heptadecyl-4,4'-bis(methylene stearate)-1,3-oxazoline	95104	901.8462	BPUYDDKNZNJELI-UHFFFAOYSA-N
Sorbitan tristearate	15181202	962.8514	IJCWFDPJFXGQBN-RYNSOKOISA-N
Tristearyl citrate	24493	948.8721	UKBHVNMEMHTWQO-UHFFFAOYSA-N
Castor oil, hydrogenated	25100	938.8150	WCOXQTXVACYMLM-UHFFFAOYSA-N
Pentaerythritol tristearate	14252002	934.8565	FWCDLNRNBHJDQB-UHFFFAOYSA-N
Sorbitan triisostearate	171343	962.8514	QWSHIYVIOOXKLL-LLPUSWRMSA-N
Sorbitan trioleate	9920343	956.8044	PRXRUNOAOLTIEF-ADSICKODSA-N
Trimethylolpropane trioleate	6436686	926.8302	BTGGRPUPMPLZNT-PGEUSFDPSA-N
Distarch glycerol	24832114	1176.4895	IQZVGYOIHLNAKB-UHFFFAOYSA-N
dioctyldodecyl adipate	3020369	706.6839	WLFITRMCTPBSQS-UHFFFAOYSA-N
Sorbitan dioleate	22833309	692.5591	TTZKGYULRVDFJJ-GIVMLJSASA-N
Distearyl citrate	11643318	696.5904	PGGUBOZIFQYBOV-UHFFFAOYSA-N
Glycerol trimyristate	11148	722.6424	DUXYWXYOBMKGIN-UHFFFAOYSA-N
Glycerol dibehenate	9831860	736.6945	GNWCZBXSKIIURR-UHFFFAOYSA-N
Tetrakis(2,4-di-tert-butyl-5-methylphenyl) [1,1'-biphenyl]-2,3-diylbis(phosphonite)	22672285	1090.7097	XMKVUPOWKZQBDQ-UHFFFAOYSA-N
Hexanoic acid, 3,5,5-trimethyl-, 1,1-[oxybis[2,2-bis[[(3,5,5-trimethyl-1-oxohexyl)oxy]methyl]-3,1-propanediyl]] ester	90684455	1094.8572	GJIDQGCYINNRBJ-UHFFFAOYSA-N
Hydroxylated lecithin	57508518	821.6146	XSEOYPMPHHCUBN-FGYWBSQSSA-N
alpha-D-Glucopyranoside, beta-D-fructofuranosyl, dioctadecanoate	5360827	874.6381	MZNXRHOLDWQYRX-CBKJUIDTSA-N
diester of 3-(laurylthio)propionic acid with 4,4'-[thiobis(2-tert-butyl-5-methylphenol)]	105368	870.5688	MILWQXYAFKWZBP-UHFFFAOYSA-N
Glycerol tripalmitate	11147	806.7363	PVNIQBQSYATKKL-UHFFFAOYSA-N
1-Octadecanaminium, N-ethyl-N,N-dioctadecyl-, ethyl sulfate	106121	927.9016	MGUHJFIMPPINPG-UHFFFAOYSA-M
N,N'-[ethylenebis(iminoethylene)]bisbehenamide	44151217	790.8003	SFBNWBOCWQUMEJ-UHFFFAOYSA-N

Glycerol trioleate	5497163	884.7833	PHYFQTYBJUILEZ-IUPFWZBJSA-N
Tristearyl phosphate	74962	854.8220	FDGZUBKNYGBWHI-UHFFFAOYSA-N
1-(Hexadecanoyloxy)-3-(octadecanoyloxy)propan-2-yl octadec-9-enoate	53422263	860.7833	QXPXMOHHFYONAC-UHFFFAOYSA-N
Sorbitan, trihexadecanoate	171319	878.7575	NVANJYGRGNEULT-BDZGGURLSA-N
Trioctadecyl phosphite	248442	838.8271	CNUJLMSKURPSHE-UHFFFAOYSA-N
Pyrrolo[3,4-c]pyrrole-1,4-dione, 2,5-dihydro-3,6-bis[4-(octadecylthio)phenyl]-1	135565960	856.5974	OECIMFUOKDGJQO-UHFFFAOYSA-N
Fatty acids, C18-unsatd., trimers	6437702	800.4077	CFQZKFWQLAHGSL-FNTYJUCDSA-N
Sucrose octabenzoate	25113553	1174.3259	AKIVKIDZMLQJCH-KWOGCLBWSA-N
beta-Cyclodextrin	444041	1134.3698	WHGYBXFWUBPSRW-FOUAGVGXSA-N
2,2-Bis(((2-cyano-3,3-diphenylacryloyl)oxy)methyl)propane-1,3-diyl bis(2-cyano-3,3-diphenylacrylate)	16134382	1060.3472	CVSXFBFIOUYODT-UHFFFAOYSA-N
Distearyl thiodipropionate	12738	682.5934	PWWSSIYVTQUJQQ-UHFFFAOYSA-N
Glycerol tristearate	11146	890.8302	DCXXMTOCNZCJGO-UHFFFAOYSA-N
Triisodecyl tridecyl trimellitic ester	3085422	672.5329	YNKHAYUWCVQHBA-UHFFFAOYSA-N
1,2,3-Propanetriol, homopolymer, (Z)-9-octadecenoate	9963243	1022.6237	NPTLAYTZMHJJDP-KTKRTIGZSA-N
Distearyl adipate	70706	650.6213	GYFBKUFUJKHFLZ-UHFFFAOYSA-N
Pentaerythritol decanoate, Decanoic acid, 2,2-bis[[(1-oxodecyl)oxy]methyl]-1,3-propanediylester	83733	752.6166	MXNODNKXIIQMMI-UHFFFAOYSA-N

Supplemental Materials and Methods.

Calculation of Molecular Descriptors. The first descriptor dataset was calculated using alvaDesc v.2.0.4 within OCHEM, which contains 5666 descriptors including constitutional, topological, charge, and geometrical descriptors. The second descriptor dataset was calculated using CDK v2.3 from OCHEM, which contains 256 constitutional, topological, geometric, electronic and hybrid descriptors (the CDK descriptors were used for the prediction of CCS values in the AllCCS webserver[8]). The third descriptor dataset contains 196 RDKit descriptors calculated using ChemDes (RDKit descriptors were used in the development of the CCSondemand prediction tool[9]).

River Water Treatment. All the water sample was filtered by 0.7 glass fiber filter, 100 mL of aliquots were passed through the solid phase extraction (SPE) (Oasis HLB cartridge, 6cc/200mg, Waters Corp.), previously conditioned with 10 mL of methanol and 10 mL of water. The SPE cartridge dried for 10 mins and was eluted with 12 mL of methanol, the filtrate was evaporated to dryness at 45 ºC under a gentle stream of N_2, the residue was redissolved in 1.5 mL of methanol and analyzed by Vion IMS-QTof (Waters, Manchester, UK), this treatment was performed in triplicates.

Conditions of Vion IMS-QTOF. The chromatographic separation was performed using a CORTECS C18 column (2.1 × 100 mm, 1.6 µm particle size, 90 Å pore size) at a flow rate of 0.3 mL min^{-1}. Mobile phases were water (A) and methanol (B), both acidified with 0.1% of formic acid (v/v). The initial proportion of B was 5%, increased to 100% over 7 minutes, kept at 100% from 7 to 11 minutes, decreased to 5% over 0.1 minutes and re-conditioned until 13 minutes.

Data were acquired on the mass spectrometer in positive mode over the mass range of 50-1000 *m/z* with a scan time of 0.2 s. Electrospray ionization (ESI) conditions were as follows: capillary voltage, 1 kV; cone voltage, 30 V; source temperature, 120 ºC; desolvation temperature, 500 ºC; cone gas flow, 50 L h^{-1}; desolvation gas flow, 800 L h^{-1}. Data were acquired in high definition MS^E mode, with the instrument was switching between two collision energy states (low energy: 6 eV, high energy ramp: 20-40 eV) in order to obtain precursor and fragment ions within a single acquisition. Leucine-Enkephalin ($[M+H]^+$, *m/z* 556.2766) at a concentration of 100 ng/mL was infused at a rate of 15 µL/min for real-time mass correction. IM separations were performed with a travelling wave velocity of 250 m/s and IMS pulse height of 45 V, N_2 was used as the drift gas at a flow of 25 mL/min. The Vion platform works at a room temperature of 25 ºC.

Supplemental Results and Discussion

Three possible sources of CCS deviations. (1) Studies have shown that while the deviations of CCS values measured on traveling wave (TWIMS) devices from different laboratories are generally less than 2%, in some cases the deviations can be at either extreme of the acceptable range leading to a higher RSD value.

(2) Presence of protomers. Isobaric protomers have been identified for some compounds due to molecules having multiple protonation sites.[5, 10] If a charged isomer pair is sufficiently resolved by IMS, different CCS values will be assigned to each isomer. Most pesticides contain multiple protonation sites in their structure, the presence of amine and carbonyl oxygen groups, for example. Two CCS values, 179.6 Å^2 and 191.21 Å^2, have been reported for marbofloxacin,[2, 3] both protomers were detected by a cyclic ion mobility systems in McCullagh et al. (2018).[10] Warnke et al. (2015)[11] have shown that the use of methanol can favor the protonation of carbonyl oxygen while acetonitrile can favor the protonation of the amine. In their work Regueiro et al. (2016)[4] used acetonitrile and water as the mobile phase, which might explain the different CCS values of fenpyroximate (205.3

Å^2 versus 215.9 Å^2), picoxystrobin (194.5 Å^2 versus 177.5 Å^2) and oxadixyl (178.3 Å^2 versus 158.8 Å^2).

(3) Inconsistent CCS calibration across different instrument systems. The CCS values of 16 compounds measured by Bijlsma et al. (2017)[2] were lower than those measured by Celma et al. (2020)[3] and Regueiro et al. (2016),[4] (see Table S2). This consistent difference might imply that the TWIMS was calibrated using a different set of standards or by considering a different set of reference points.

Partially orthogonal molecular information provided by CCS. As CCS is related to the size, shape, and charge of gas-phase ions, it is understandable that CCS is highly correlated to *m/z* values. However, distinct correlations between CCS and *m/z* have been observed for the compounds that possess different structural characteristics.[6, 12] Belova et al. showed that the plasticizers, organophosphate flame retardants and per- and polyfluoroalkyl substances (PFAS) present different CCS versus *m/z* trendlines.[6] PFAS contain carbon-fluorine bonds, and their CCS values are much lower in general than other compounds of similar *m/z* values. Hines et al. present that most 1440 CCS values of drugs and drug-like compounds fall within ±10% threshold, the compounds with *m/z* 300-350 possess CCS values ranging from 150 to 210 Å^2.[12] These studies proved that besides the mass of molecules, the molecular shape and compactness can also affect the CCS values. The different CCS values of molecules at a given *m/z* value indicate that CCS can provide partially orthogonal molecular information for features in targeted and untargeted screening analysis.

Reasons leading to high prediction errors. Among the 22 protonated molecules for which the errors in the predicted values were more than 5%, there were some pesticide and drug compounds that had structures which exhibited multiple possible protonation sites. For example, two different CCS values (160.5 Å^2 and 176.2 Å^2) have been reported for acetopromazine in previous studies,[2, 3] and protonation can occur on the carbonyl oxygen or the aminic group for this molecule. The high prediction error (8.9%) for glycocholic acid (measured 187.6 Å^2/predicted 207.8 Å^2) may also be due to the presence of multiple protomers. A $^{TW}CCS_{N2}$ value of 205.2 Å^2 for protonated glycocholic acid, was obtained in Hines et al.,[12] which matched well with our predicted CCS value. A predicted CCS value of 179.89 Å^2 was obtained for this compound, which matched well with the CCS value of the more extended protomer. The high prediction error (8.9%) for glycocholic acid (measured 187.6 Å^2/predicted 207.8 Å^2) may also be due to the presence of multiple protomers. A $^{TW}CCS_{N2}$ value of 205.2 Å^2 for protonated glycocholic acid, was obtained in Hines et al.,[12] which matched well with our predicted CCS value.

Similar behavior was also observed in the work of Zhou et al.,[13] in which the predicted CCS value (172.3 Å2) for S-methyl-5'-thioadenosine matched well with CCS of the more extended protomer (170.9 Å^2) compared to the CCS of the more compact protomer (162.5 Å^2). One possible explanation for this phenomenon is that for the compact protomers, the protons are trapped in the core of the molecules and do not increase the overall size of the molecule. Thus, slightly lower experimental CCS values are obtained which can lead to relatively higher prediction errors. The less accurate CCS prediction for doramectin (measured 308.7 Å^2/predicted 284.7 Å^2), N,N'-Ethylenebis(stearamide) (measured 280.5 Å^2/predicted 296.6 Å^2), may be attributed to the low number of similar chemical structures in the training set.

Comparison Between the SVM Model and Public CCS Prediction Tools. The comparison between the SVM model and public CCS prediction tools is shown in Figure S6. Although

CCSondemand provides more accurate predicted CCS values, currently, it cannot accurately predict the CCS of silicon-containing molecules, such as (3-aminopropyl) triethoxysilane (measured 153.7 Å^2/predicted 121.1 Å^2) and flusilazole (measured 174.3 Å^2/predicted 107.4 Å^2). In CCSondemand such molecules cleave at the site of silicon and only a part of structure is considered, thus introducing a bias towards the prediction of lower-than-expected CCS values.

The heat-map in Figure S7 shows that SVM provides more accurate CCS predictions for the $[M+H]^+$ adduct of lipid and lipid-like molecules, organic nitrogen compounds and organic oxygen compounds, when compared against the other models. The prediction of CCS values by the SVM model for benzenoids, which are common class of molecules found in plastics, is also acceptable, with a MRE of 1.6%. The SVM also outperforms the other tools in the prediction of CCS values for the $[M+Na]^+$ adduct for benzenoids, organic acids and derivatives and organoheterocyclic compounds, all of which have an MRE of less than 2%. The relatively high prediction errors for $[M+Na]^+$ adducts of organic nitrogen compounds and organic oxygen compounds may be due to the low numbers of experimental CCS values available in these two super classes. Organic nitrogen compounds account for only 1.2% (8/645) of the CCS values available for $[M+Na]^+$ adducts and organic oxygen compounds for 3.6% (23/645). The same reason can be used to explain the high MRE values for $[M+H]^+$ adducts of organic nitrogen compounds and organosulfur compounds, which only account for 2.9% and 0.5% of the experimental CCS value available for $[M+H]^+$ adducts. This highlights the need for collecting more experimental CCS values from a range of compound classes.

Weighting of CDK descriptors. The 20 most important CDK descriptors for the prediction of the CCS value of the $[M+H]^+$ adducts in XGBoost model, are shown in Figure S10, a brief introduction of these descriptors is given in Table S11. The Atomic and Bond Contributions of Van der Waals volume (VABC) was found to be the most important CDK descriptor for the prediction of CCS values. CCS was strongly correlated with VABC, as shown in Figure S11, with the linear regression line having an R^2 value of 0.9533. VABC is a molecular volume property, which is estimated by the atomic contributions and the number of atoms, bonds, and rings.[14] Molecular connectivity chi indices: VP.0, VP.1, SP.1 and SP.2 were also found to be influential CDK descriptors, each chi index is a sum of weighted subgraphs in which the weights are functions of the molecular connectivity delta values.[15] Molecular connectivity chi indices is one type of topological indices, which were previously used to predict the retention time of small molecules.[16] Atom molar refractivity (AMR) is a constitutive-additive property, as described by Ghose and Crippen,[17] it represents the volume of molecules.[18] Atomic polarizability (apol) calculates the sum of the atomic polarizabilities (including implicit hydrogens) and bpol calculates the sum of the absolute value of the difference between atomic polarizabilities of all bonded atoms in the molecule. AMR and apol were used to predict CCS values by Zhou et al. (2016).[13] The Weiner path number (WPATH) is a topological descriptor, which is calculated as the sum of the lengths of the shortest paths between all pairs of vertices in the chemical graphs.[19] Eccentric connectivity index (ECCEN) is a topological descriptor, it considers the eccentricity and valency of each vertex involved in a molecular graph. Its calculation can be performed from the distance matrix of a hydrogen-suppressed molecular graph after the vertices have been numbered arbitrarily.[20] The first kappa shape index (Kier1) is a also a topological property, it characterize the molecular shape,[21] kappa index was used for predicting the CCS values in AllCCS.[8] The nAtom and MW are constitutional descriptors, which measure the total

number of atoms and molecular weight of molecules. Other important descriptors are provided in Table S11. The analysis of MDs weights substantiates the correlation of the CCS of a molecule to the size and shape of that ionized molecule.

Applicability of our CCS prediction model. In order to evaluate whether out models can be used to predict the CCS values of molecules in CPPdb and FCCdb, we compared the chemical space covered by our collected CCS records to that of CPPdb and FCCdb, the results are shown in Figure S13. A large proportion of molecules in CPPdb and FCCdb was covered by the chemical space of our collected CCS records. However, there are still many molecules which are out of this chemical space, we carefully examined these kinds of molecules in order to find their structural characteristics. Table S12 presents 50 compounds that locate far from the group center, generally, many of these molecules have relatively high molecular weights (MW), their MW range from 650 to 1200 Da. However, most compounds in our CCS dataset have MW ranging from 150 to 600 Da (see Figure 1).

In addition to their high MW, these compounds appear to have linear-chain molecular structures, such as polyoxyethylene (23) lauryl ether and cetyl poly(oxyethylene) ether, these compounds are alkyl PEG ethers, which are normally used as non-ionic surfactants in plastics.[22] Alkyl glycerol esters, such as glyceryl tribehenate, glyceryl tri(12-acetoxystearate), glycerol trimyristate and glycerol dibehenate, were also included, these compounds are normally used as antistatic agents.[23] Other compounds include emulsifier in plastics, such as sorbitan trioleate, sorbitan dioleate and sorbitan trihexadecanoate; plasticizers: tristearyl citrate, distearyl citrate, dioctyldodecyl adipate; antioxidant: distearyl thiodipropionate, trioctadecyl phosphite; organophosphate flame retardant: tristearyl phosphate. These compounds contain long alkyl chains in their structures and can undergo more collisions with drift gas when passing through the drift cell. The comparison of the chemical space between our collected CCS dataset and CPPdb, FCCdb highlights that more compounds with high molecular mass and linear-chain structure should be incorporated into CCS dataset.

References:

1. Song, X. C.; Dreolin, N.; Damiani, T.; Canellas, E.; Nerin, C., Prediction of Collision Cross Section Values: Application to Non-Intentionally Added Substance Identification in Food Contact Materials. *J. Agric. Food Chem.* **2022,** *70*, (4), 1272-1281.
2. Bijlsma, L.; Bade, R.; Celma, A.; Mullin, L.; Cleland, G.; Stead, S.; Hernandez, F.; Sancho, J. V., Prediction of Collision Cross-Section Values for Small Molecules: Application to Pesticide Residue Analysis. *Anal. Chem.* **2017,** *89*, (12), 6583-6589.
3. Celma, A.; Sancho, J. V.; Schymanski, E. L.; Fabregat-Safont, D.; Ibanez, M.; Goshawk, J.; Barknowitz, G.; Hernandez, F.; Bijlsma, L., Improving Target and Suspect Screening High-Resolution Mass Spectrometry Workflows in Environmental Analysis by Ion Mobility Separation. *Environ. Sci. Technol.* **2020,** *54*, (23), 15120-15131.
4. Regueiro, J.; Negreira, N.; Berntssen, M. H., Ion-Mobility-Derived Collision Cross Section as an Additional Identification Point for Multiresidue Screening of Pesticides in Fish Feed. *Anal. Chem.* **2016,** *88*, (22), 11169-11177.
5. Hinnenkamp, V.; Klein, J.; Meckelmann, S. W.; Balsaa, P.; Schmidt, T. C.; Schmitz, O. J., Comparison of CCS Values Determined by Traveling Wave Ion Mobility Mass Spectrometry and Drift Tube Ion Mobility Mass Spectrometry. *Anal. Chem.* **2018,** *90*, (20), 12042-12050.
6. Belova, L.; Caballero-Casero, N.; van Nuijs, A. L. N.; Covaci, A., Ion Mobility-High-Resolution Mass Spectrometry (IM-HRMS) for the Analysis of Contaminants of Emerging Concern (CECs): Database Compilation and Application to Urine Samples. *Anal. Chem.* **2021,** *93*, (16), 6428-6436.
7. Mullin, L.; Jobst, K.; DiLorenzo, R. A.; Plumb, R.; Reiner, E. J.; Yeung, L. W. Y.; Jogsten, I. E., Liquid chromatography-ion mobility-high resolution mass spectrometry for analysis of pollutants in indoor dust: Identification and predictive capabilities. *Anal. Chim. Acta* **2020,** *1125*, 29-40.
8. Zhou, Z.; Luo, M.; Chen, X.; Yin, Y.; Xiong, X.; Wang, R.; Zhu, Z. J., Ion mobility collision cross-section atlas for known and unknown metabolite annotation in untargeted metabolomics. *Nat. Commun.* **2020,** *11*, (1), 4334.
9. Broeckling, C. D.; Yao, L.; Isaac, G.; Gioioso, M.; Ianchis, V.; Vissers, J. P. C., Application of Predicted Collisional Cross Section to Metabolome Databases to Probabilistically Describe the Current and Future Ion Mobility Mass Spectrometry. *J. Am. Soc. Mass Spectrom.* **2021,** *32*, (3), 661-669.
10. McCullagh, M.; Giles, K.; Richardson, K.; Stead, S.; Palmer, M., Investigations into the performance of travelling wave enabled conventional and cyclic ion mobility systems to characterise protomers of fluoroquinolone antibiotic residues. *Rapid Commun. Mass Spectrom.* **2019,** *33 Suppl 2*, 11-21.
11. Warnke, S.; Seo, J.; Boschmans, J.; Sobott, F.; Scrivens, J. H.; Bleiholder, C.; Bowers, M. T.; Gewinner, S.; Schollkopf, W.; Pagel, K.; von Helden, G., Protomers of benzocaine: solvent and permittivity dependence. *J. Am. Chem. Soc.* **2015,** *137*, (12), 4236-4242.
12. Hines, K. M.; Ross, D. H.; Davidson, K. L.; Bush, M. F.; Xu, L., Large-Scale Structural Characterization of Drug and Drug-Like Compounds by High-Throughput Ion Mobility-Mass Spectrometry. *Anal. Chem.* **2017,** *89*, (17), 9023-9030.
13. Zhou, Z.; Shen, X.; Tu, J.; Zhu, Z. J., Large-Scale Prediction of Collision Cross-Section Values for Metabolites in Ion Mobility-Mass Spectrometry. *Anal. Chem.* **2016,** *88*, (22), 11084-11091.

14. Zhao, Y. H.; Abraham, M. H.; Zissimos, A. M., Fast Calculation of van der Waals Volume as a Sum of Atomic and Bond Contributions and Its Application to Drug Compounds. *J. Org. Chem.* **2003,** *68*, (19), 7368-7373.
15. Devillers, J.; Balaban, A. T., Topological Indices and Related Descriptors in QSAR and QSPR, 1999.
16. Aćimović, M.; Pezo, L.; Tešević, V.; Čabarkapa, I.; Todosijević, M., QSRR Model for predicting retention indices of Satureja kitaibelii Wierzb. ex Heuff. essential oil composition. *Ind. Crop. Prod.* **2020,** *154*, 112752.
17. Ghose, A. K.; Crippen, G. M., Atomic physicochemical parameters for three-dimensional-structure-directed quantitative structure-activity relationships. 2. Modeling dispersive and hydrophobic interactions. *J. Chem. Inf. Comput. Sci.* **1987,** *27*, (1), 21-35.
18. Padron, J.; Carrasco, R.; Pellon, R., Molecular descriptor based on a molar refractivity partition using Randic-type graph-theoretical invariant. *J. Pharm. Pharmaceut. Sci.* **2002,** *5*, (3), 258-266.
19. Rouvray, D. H.; King, R. B., *Topology in chemistry: Discrete mathematics of molecules*. Elsevier: 2002.
20. Sharma, V.; Goswami, R.; Madan, A. K., Eccentric Connectivity Index: A Novel Highly Discriminating Topological Descriptor for Structure−Property and Structure−Activity Studies. *J. Chem. Inf. Comput. Sci.* **1997,** *37*, (2), 273-282.
21. Hall, L. H.; Kier, L. B., *The Molecular Connectivity Chi Indexes and Kappa Shape Indexes in Structure-Property Modeling*. John Wiley & Sons, Ltd.,: 2007; Vol. 2.
22. Cowan-Ellsberry, C.; Belanger, S.; Dorn, P.; Dyer, S.; McAvoy, D.; Sanderson, H.; Versteeg, D.; Ferrer, D.; Stanton, K., Environmental Safety of the Use of Major Surfactant Classes in North America. *Crit. Rev. Environ. Sci. Technol.* **2014,** *44*, (17), 1893-1993.
23. Hu, Y.; Du, Z.; Sun, X.; Ma, X.; Song, J.; Sui, H.; Debrah, A. A., Non-targeted analysis and risk assessment of non-volatile compounds in polyamide food contact materials. *Food Chem.* **2021,** *345*, 128625.
